# Effects of Fluoride Long-Term Exposure over the Cerebellum: Global Proteomic Profile, Oxidative Biochemistry, Cell Density, and Motor Behavior Evaluation

**DOI:** 10.3390/ijms21197297

**Published:** 2020-10-02

**Authors:** Géssica Oliveira Lopes, Maria Karolina Martins Ferreira, Lodinikki Davis, Leonardo Oliveira Bittencourt, Walessa Alana Bragança Aragão, Aline Dionizio, Marília Afonso Rabelo Buzalaf, Maria Elena Crespo-Lopez, Cristiane Socorro Ferraz Maia, Rafael Rodrigues Lima

**Affiliations:** 1 Laboratory of Functional and Structural Biology, Institute of Biological Sciences, Federal University of Pará, Belém, PA 66075-110, Brazil; gessicalopes_22@hotmail.com (G.O.L.); krolmarrtins93@gmail.com (M.K.M.F.); lodinikkidavis@hotmail.com (L.D.); leo.bittencourt25@gmail.com (L.O.B.); walessa.aragao@gmail.com (W.A.B.A.); 2 Bauru School of Dentistry, Department of Biological Sciences, University of São Paulo, Bauru, SP 17012-90, Brazil; alinesdionizio@usp.br (A.D.); mbuzalaf@fob.usp.br (M.A.R.B.); 3 Laboratory of Molecular Pharmacology, Institute of Biological Sciences, Federal University of Pará, Belém, PA 66075-110, Brazil; maria.elena.crespo.lopez@gmail.com; 4 Laboratory of Inflammation and Behavior Pharmacology, Pharmacy Faculty, Institute of Health Science, Federal University of Pará, Belém, PA 66075-110, Brazil; crismaia@ufpa.br

**Keywords:** fluoride, cerebellum, oxidative stress, proteomic

## Abstract

Although the literature does not provide evidence of health risks from exposure to fluoride (F) in therapeutic doses, questions remain about the effects of long-term and high-dose use on the function of the central nervous system. The objective of this study was to investigate the effect of long-term exposure to F at levels similar to those found in areas of artificial water fluoridation and in areas of endemic fluorosis on biochemical, proteomic, cell density, and functional parameters associated with the cerebellum. For this, mice were exposed to water containing 10 mg F/L or 50 mg F/L (as sodium fluoride) for 60 days. After the exposure period, the animals were submitted to motor tests and the cerebellum was evaluated for fluoride levels, antioxidant capacity against peroxyl radicals (ACAP), lipid peroxidation (MDA), and nitrite levels (NO). The proteomic profile and morphological integrity were also evaluated. The results showed that the 10 mg F/L dose was able to decrease the ACAP levels, and the animals exposed to 50 mg F/L presented lower levels of ACAP and higher levels of MDA and NO. The cerebellar proteomic profile in both groups was modulated, highlighting proteins related to the antioxidant system, energy production, and cell death, however no neuronal density change in cerebellum was observed. Functionally, the horizontal exploratory activity of both exposed groups was impaired, while only the 50 mg F/L group showed significant changes in postural stability. No motor coordination and balance impairments were observed in both groups. Our results suggest that fluoride may impair the cerebellar oxidative biochemistry, which is associated with the proteomic modulation and, although no morphological impairment was observed, only the highest concentration of fluoride was able to impair some cerebellar motor functions.

## 1. Introduction

Fluoride is naturally present in the earth’s crust and is frequent in a variety of environments [1]. However, this ion does not exist in an isolated form, always being associated with other elements constituting fluoridated compounds, which are found in rock minerals and volcanic soil [2]. Water is one of the main sources of systemic exposure to fluoride. This ion can be naturally present in the water, due to the geological sources, or can be artificially added to public water distribution systems, with anticariogenic purposes, at controlled concentrations [2].

According to the recommendations of the World Health Organization (WHO), the optimal fluoride concentration in supply waters is in the range of 0.5–1 mg/L, which can be adjusted between 0.7–1.2 mg/L depending on the mean local temperature [3,4]. Besides that, it is important to consider that some regions naturally present high levels of fluoride for natural or anthropogenic reasons; in this way, it is necessary to control the artificial fluoridation levels in order to assure efficacy for caries control and safety [5].

Optimal water fluoridation is not associated with other harmful effects besides dental fluorosis [6]. On the other hand, higher concentrations might also affect bone [7], liver [8], kidney [9,10], and gut [11,12]. In addition, studies have suggested that ingesting high doses of fluoride during childhood may influence on intellectual abilities and cognitive development [13,14,15,16], although there are no reports of harmful effects on humans exposed to therapeutic doses [17].

The possible mechanisms by which fluoride damages the structures associated with cognitive functions have been reported in the literature [18,19,20]. However, little has been investigated on the effects on motor aspects and their related organs [21,22]. In this perspective, it is important to note that motor functions are generated from various structures of the central nervous system (CNS), such as motor cortex, basal ganglia, spinal cord, and cerebellum [23], among which the cerebellum is important for motor coordination and motor learning, being mainly associated with planning and fine regulation of voluntary movement, as well as balance control [24,25]. Damage to this structure can promote motor deficits [26] with important clinical repercussion.

In this context, considering that fluoride can damage the CNS, but with few reports on the possible mechanisms of damage to motor functions at high concentrations, we sought to evaluate the effects of two fluoride concentrations administered through the drinking water, equivalent to those found in the public water supply and in areas of endemic fluorosis regions, in the cerebellum of mice. We performed molecular, morphological, and functional analyses, bringing the unprecedented evaluation of the cerebellar proteome, capable of tracing possible biomarkers of exposure and damage.

## 2. Results

### 2.1. Prolonged Exposure to NaF Did Not Impair Weight Gain

**Figure 1:**
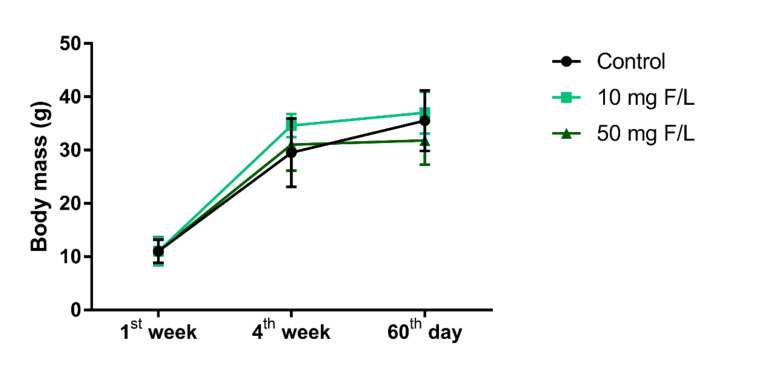
Effect of prolonged exposure to fluoride at doses of 10 mg F/L and 50 mg F/L for 60 days on mice body mass gain. *n* = 20 animals per group. The results are expressed as mean ± SEM with two-way ANOVA.

During the experimental period, the fluoride exposure at both concentrations did not impair the body weight gain as shown in Figure 1. The experimental groups showed no difference in the beginning, middle, and end of exposure protocol (*p* = 0.05).

### 2.2. Long-Term Exposure to NaF Increased Cerebellum Fluoride Levels

**Figure 2:**
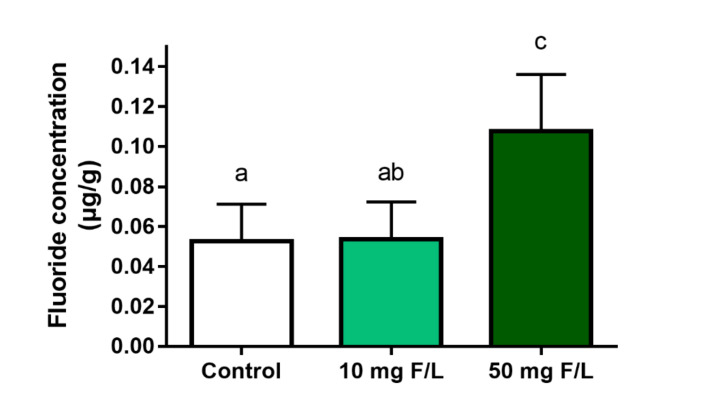
Analysis of F levels (µg/g) in the cerebellum of mice after 60 days of exposure to ultrapure water (control group) and fluoridated water (10 mg F/L e 50 mg F/L). Results are expressed as mean ± SEM. *n* = 10 animals per group. One-way ANOVA and Tukey’s post hoc test, *p* < 0.05. Similar overwritten letters did not show significant statistical differences.

We observed that the daily water consumption was approximately 5 mL per animal, equivalent to 0.05 mg F/day for 10 mg F/L group and 0.25 mg F/day for the 50 mg F/L group. In this way, the prolonged exposure to fluoride promoted significant differences in the levels of fluoride in the cerebellum of animals exposed to 50 mg F/L (0.10 ± 0.014 µg/g) compared to the control group (0.05 ± 0.01 µg/g; *p* = 0.03) and 10 mg F/L group (0.05 ± 0.009 µg/g; *p* = 0.02). However, there was no difference when comparing the 10 mg F/L and control groups (*p* > 0.05) (Figure 2).

### 2.3. Exposure to Fluoride Modulated the Cerebellar Oxidative Biochemistry Balance in Mice

**Figure 3:**
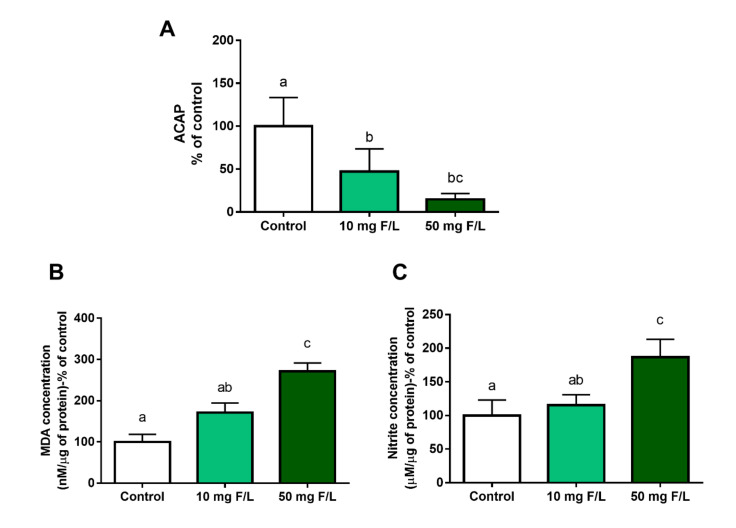
Effects of long-term exposure to NaF on oxidative biochemistry of the cerebellum of mice exposed for 60 days. The graph shows, as a percentage of the control, the results in the groups that received deionized water, and fluoride water (10 mg F/L e 50 mg F/L, respectively). In (**A**) antioxidant capacity against peroxyl radicals (ACAP) levels, (**B**) lipid peroxidation (LPO) analysis, (**C**) Nitrite concentration. *n* = 10 animals per group. One-way ANOVA followed by Tukey’s test, *p* < 0.05. Similar overwritten letters did not show significant statistical differences.

Fluoride exposure decreased ACAP levels at 10 mg F/L (47.24 ± 11.1%; *p* = 0.02) and 50 mg F/L (14.58 ± 3.42%; *p* = 0.001) groups compared to the control group (100 ± 16.7%) (Figure 3A). Additionally, LPO levels were not significantly higher in the group exposed to 10 mg F/L (171.1 ± 23.17%; *p* = 0.0924) in relation to the control group (100 ± 18.53%), presenting a significant difference only between the group exposed to 50 mg F/L (271 ± 19.77%, *p* = 0.0004) when compared to the control group (Figure 3B). In addition, the levels of nitrites increased only in the group with the highest concentration (186.9 ± 11.76%) compared to the 10 mg F/L group (115.2 ± 6.9%, *p* = 0.0009) and with the control group (100 ± 11.54%, *p* = 0.0003) (Figure 3C).

### 2.4. Prolonged Exposure to Fluoride Significantly Modulated the Cerebellar Proteome of Mice

In the proteomic analysis, 386 unique proteins were found in the control group. While in the 10 and 50 mg F/L groups, 118 and 5 unique proteins were found, respectively (see Table S1).

In the analysis of proteins with difference in expression, 262 altered proteins were found in the 10 mg F/L group compared to the control group; 252 were downregulated and 10 were upregulated (Table S2) and in the 50 mg F/L group all 235 proteins were downregulated (Table S3) compared to the control group.

When analyzing the biological processes of the 10 mg F/L group compared to the control group, we found 23 altered processes. The most significant were axon guidance (14.9%), followed by regulation of dendrite mophorgenesis (9.6%), positive regulation of DNA-binding transcription factor activity (8.8%), regulation of axion extension, positive regulation of JNK cascade (4.4%), besides other processes related to mitochondrial activity and neurotransmitter regulation.

**Figure 4:**
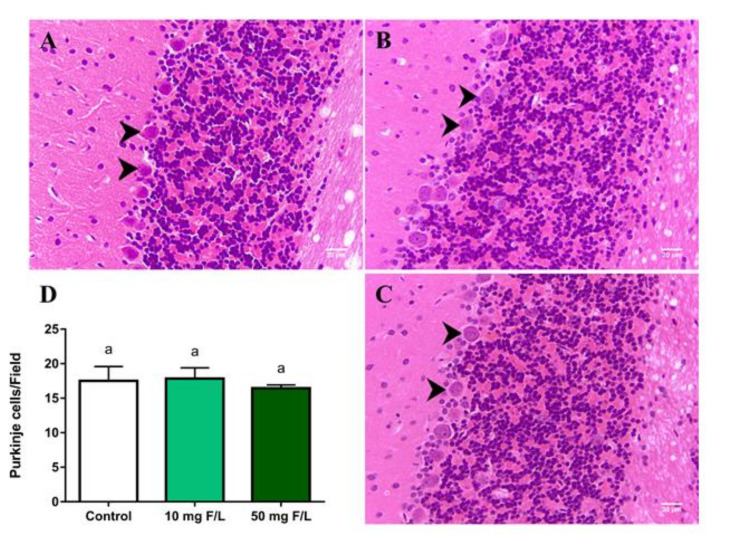
Effects of long-term exposure to NaF on Purkinje cell density in mice cerebellum (arrow). (**A**–**C**) represent photomicrographs of groups 10 mg F/L, 50 mg F/L, and control, respectively. The results are expressed as mean ± standard error of the number of cells counted per field (**D**). *n* = 10 animals per group. One-way ANOVA followed by Tukey’s test, *p* < 0.05. Scale bar: 20 μm. Similar overwritten letters did not show significant statistical differences.

In the group exposed to the highest concentration, 50 mg F/L, (Figure 4) 19 biological processes were affected, such as axon guidance (13.6%), followed by regulation of axogenesis (13.6%), regulation of release of sequestered calcium ion into cytosol (9.1%), dendritic spine morphogenesis (7.3%), and also processes involved in mitochondrial and neuronal processes (Table 1).

### 2.5. Long-Term Exposure to NaF Did Not Change the Density of Purkinje Cells

There were no changes in the density of Purkinje cells in the exposed groups 10 mg F/L (17.84 ± 0.78; *p* = 0.94) and 50 mg F/L (16.44 ± 0.24; *p* = 0.60) when compared to the control group (17.5 ± 1.05) (Figure 4).

### 2.6. Chronic Exposure to NaF at 10 and 50 mg F/L Altered Functional Locomotion Parameters, Both Horizontal and Vertical, and Exploration

**Figure 5:**
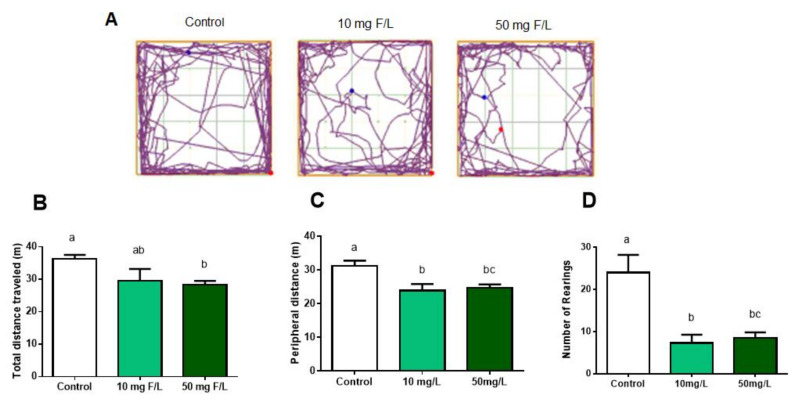
Effects of long-term exposure to NaF in mice for 60 days in horizontal and vertical locomotion. Results are expressed as mean ± SEM of: (**A**) Representation of the analysis of horizontal locomotor activity in the open field test of groups control, 10 mg F/L, 50 mg F/L, respectively. (**B**) Total distance traveled; (**C**) distance traveled at the periphery; and (**D**) number of rearing. *n* = 10 animals per group. One-way ANOVA followed by Tukey’s test, *p* < 0.05. Similar overwritten letters did not show significant statistical differences.

Regarding the number of rearing, the vertical exploration, there was a difference both in the animals exposed to 10 mg F/L (7.33 ± 1.9; *p* < 0.0001) and 50 mg F/L (8.5 ± 1.3; *p* = 0.0002), in relation to the control group (24.0 ± 4.18) (Figure 5D).

### 2.7. Chronic Exposure to NaF Did Not Impaired Motor Coordination and Animal Balance

**Figure 6:**
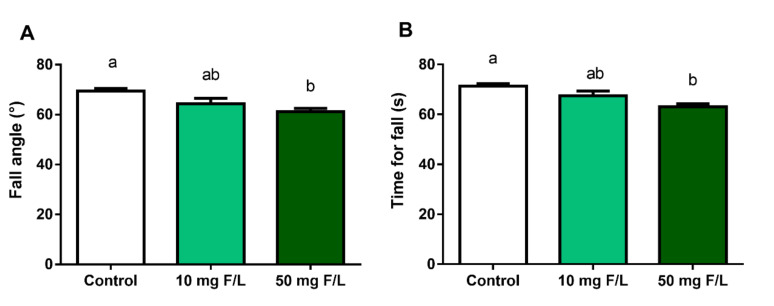
Effects of long-term exposure to NaF for 60 days on motor coordination and mice balance. Falling angle (**A**) and time to fall (**B**). Results are expressed as mean ± SEM. *n* = 10 animals per group. One-way ANOVA followed by Tukey’s test, *p* < 0.05. Similar overwritten letters did not show significant statistical differences.

The animals exposed to 10 mg F/L (64.38° ± 2.2°) did not present significant differences in relation to the drop angle when compared to the control (69.38° ± 1.1°; *p* = 0.09) when performing the inclined plane test. The animals exposed to 50 mg F/L (61.25° ± 1.25°) had significant differences when compared to the control group (69.38° ± 1.1°; *p* = 0.004) (Figure 6A). When observing the time to fall, only the 50 mg F/L group (63.13° ± 1.15°) presented a significant difference when compared to the control group (71.38° ± 0.9°; *p* = 0.001), with no significant differences between the 10 mg F/L group (67° ± 1.9°) and the control group (*p* > 0.05) (Figure 6B).

### 2.8. Exposure to NaF, at Both Concentrations, Did Not Change Locomotor Activity

**Figure 7:**
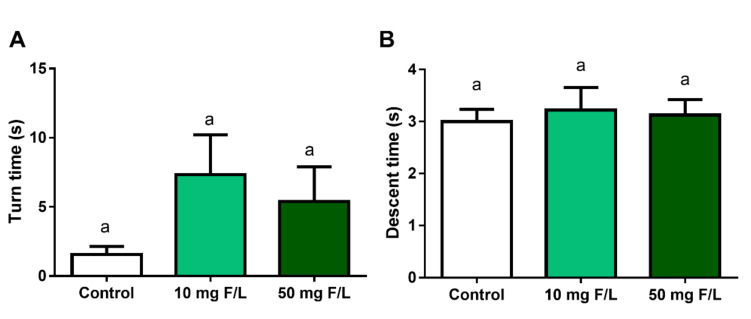
Effects of long-term exposure to NaF on the evaluation of lower limb movement disorders in mice. The graph shows the turning time (**A**) and descending time (**B**) on the vertical beam for the control groups, 10 mg F/L and 50 mg F/L. The results are expressed as mean ± SEM. *n* = 10 animals per group. One-way ANOVA followed by Tukey’s test *p* < 0.05. Similar overwritten letters did not show significant statistical differences.

Long-term exposure to NaF did not alter the results of the pole test. At the turnaround time, there was no significant difference between the 10 mg F/L group (7.3 ± 2.8 s) and the control group (1.5 ± 0.57 s; *p* > 0.05). There was also no significant difference between the 50 mg F/L (5.3 ± 2.5 s) when compared to the control group (*p* > 0.05) (Figure 7A).

In the descent time parameter, the 10 mg F/L group (3.2 ± 0.43 s) took a similar time to descend the vertical beam when compared to the control group (3.0 ± 0.23 s; *p* > 0.05) Likewise, there was no significant difference between the descent time between the control animals and those treated with 50 mg F/L (3.12 ± 0.29 s) (*p* > 0.05) (Figure 7B).

### 2.9. There Was No Change in Motor Balance in Animals Exposed to NaF

**Figure 8:**
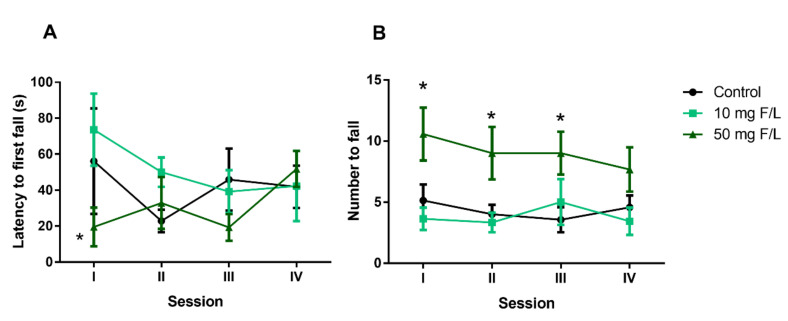
Effects of long-term exposure to NaF in forced motor motion of mice. The graph shows the latency time (**A**) and fall numbers (**B**), evaluated in the Rotarod apparatus. The results are expressed as mean ± SEM. *n* = 10 animals per group. Two-way ANOVA followed by Tukey’s test, * *p* < 0.05.

Long-term exposure to NaF changed the latency time at first exposure in the 50 mg F/L group (30.86 ± 7.6 s) compared to the 10 mg F/L group (51.27 ± 7.7 s) (*p* = 0.03). There was no difference in first exposure between the control group (41.67 ± 6.9 s) and 10 mg F/L (51.27 ± 7.7 s; *p* > 0.05). In exposures II, III, and IV, the latency was restored, showing no statistical difference between groups (*p* > 0.05) (Figure 8A). The 50 mg F/L group (9.06 ± 0.5) showed an increase in the number of falls in the first three exposures compared to the 10 mg F/L group (3.8 ± 0.38) (*p* = 0.0160) and control group (4.3 ± 0.34; *p* = 0.0442). At the fourth exposure, there was no difference in the number of falls between groups (*p* > 0.05) (Figure 8B).

## 3. Discussion

Our results show the exposure to 50 mg F/L had a greater capacity to promote biochemical, proteomic, and functional changes in the cerebellum compared to exposure to 10 mg F/L. The changes observed among the groups are demonstrated by an increase in fluoride levels, with a decrease in ACAP and an increase in MDA and NO levels in animals exposed to 50 mg F/L while the group exposed to 10 mg F/L showed only reduced ACAP. Regarding the proteomic profile, changes were observed in both groups, without, however, being observed a reduction in the density of Purkinje cells of the exposed animals. Our motor tests revealed that exposure to 50 mg F/L resulted in impaired animals’ motor skills, demonstrating, for the first time, the cerebellar susceptibility to high fluoride concentrations.

In this study, two doses were used, 10 and 50 mg F/L, established in the literature for inducing plasma fluoride levels in rodents equivalent to those observed in populations exposed to artificially fluoridated water and those living in endemic regions of fluorosis, respectively [12,27,28]. Thus, it is possible to compare these exposure models with the values obtained by humans daily in both circumstances [12].

The fluoride absorption occurs mainly through the gastrointestinal tract by non-ionic diffusion [29,30]. It is pH-dependent, so the lower the stomach pH, the greater the amount absorbed. Its high electronegativity allows the conversion of the fluoride ion into hydrogen fluoride (HF), facilitating its cellular interaction and distribution through the bloodstream [2,31,32]. The ability to cross different types of cell membranes, including the blood–brain barrier, is conFigured as one of its toxicity mechanisms, being related to changes in the metabolism and physiology of neurons and glia, modifying the memory and learning process of individuals [20,30,33]. Our data demonstrate that fluoride at high concentrations is able to cross the cerebellar blood–brain barrier, once the fluoride levels observed in the tissue of animals exposed to the concentration of 50 mg F/L were significantly higher than those found in control animals.

The cellular effects from exposure to fluoride depends on the time, concentration, and cell type and its toxicity is closely related to this characteristic. Depending on the cell type, it can act as an enzyme inhibitor or stimulator. Some studies demonstrate cytotoxic changes in different signaling pathways involved in cell proliferation and apoptosis, such as mitogen-activated protein kinases (MAPK), p53, activator protein 1 (AP 1), and nuclear factor Kappa B (NF-κB) [34,35,36].

Among the cellular toxicity mechanisms of fluoride there is oxidative stress [37,38]. This event is featured by an increase in reactive oxygen species (ROS) to the detriment of antioxidant cell response. This mechanism promotes the oxidation of various biomolecules such as lipids, proteins, and DNA [39]. In our analyses, we observed an increase in LPO and NO in the group exposed to 50 mg F/L and the reduction of antioxidant capacity in both exposure groups, which reveals the occurrence of an imbalance in the cellular redox system, suggesting the presence of oxidative stress. These finding corroborate with the data in the literature about the fluoride ability to promote ROS generation and changes in cellular antioxidant defenses [2,37,38].

In this way, the oxidative stress mechanism is one of the main pathways of fluoride toxicity, as it acts on the biochemical imbalance promoting changes in the physiological functions of the cell [30]. As an example, there is the interaction of fluoride ions with amino acid chains of enzymes associated with mitochondrial function such as glycolytic enzymes in the Krebs’ cycle, which leads to a functional inhibition, hence contributing to the generation of ROS [40]. In addition, the Na^+^/K^+^ -ATPases enzymes that are responsible for maintaining the membrane potential in mitochondria, are also sensitive to the action of fluorine and may suffer functional inhibition resulting in the reduction of ATP [41] and alteration in cell permeability, consequently causing the inhibition of cell respiration [42]. These mitochondrial changes lead to a higher generation of ROS that induces the action of cellular antioxidant compounds, however if this level of ROS is too high, cells tend not to be able to intercept these compounds resulting in oxidative damage [43]. Furthermore, the antioxidant system is also the target of the toxic action of fluoride as evidenced in some previous studies [37,38,44,45,46].

Among the consequences of oxidative stress, we can highlight the lipid peroxidation, in which the oxidation of polyunsaturated fatty acids in cell membranes occurs by the action of ROS [47]. One of the metabolites of this reaction is MDA, which is the marker analyzed in this study. Therefore, we observed the presence of LPO in the group exposed to a dose of 50 mg F/L, as a result of which we can understand that exposure to fluoride promotes molecular damage that leads to changes in the permeability and stability of lipid membranes [48].

The redox imbalance is able to modulate most of the macromolecules, generating changes at the nuclear, protein and metabolic levels. In our study, observing the proteomic profile, we found several changes in protein regulation; among them, the modulation of several cytochrome C subunits, such as subunit 1 (Q9CZ13, Q9CZ13, Q9CZ13), subunit 2 (Q9DB77, P00405), subunit 4 (P19783), and subunit 5A (P12787) in both fluoride exposed groups. In the group exposed to 10 mg F/L, there were alterations in subunits 6A (P43024), cytochrome c somatic (P62897) and cytochrome c1 heme protein (Q9D0M3), and in the group 50 mg F/L, in cytochrome c oxidase subunit NDUFA4 (Q62425). These proteins, which are related to transport in the respiratory electron chain of mitochondria, are closely related to energy production and are also involved in cell death signaling pathways [49,50].

In addition, changes were also found in the subunits of ATP synthase, such as subunit B1 (Q9CQQ7), alpha (Q03265), beta (P56480), subunit d (Q9DCX2), and subunit O (Q9DB20) in both groups, 10 mg F/L and 50 mg F/L, and subunits delta (Q9D3D9) and f (P56135) only in 10 mg F/L group. These proteins are enzymatic complexes in the mitochondrial membrane that are important for generating energy through the transport of hydrogen molecules across the cell membrane [51].

Mitochondria are essential organelles for the function of cellular respiration and energy generation in aerobic living beings and changes in their metabolism will consequently modulate the production of energy and increase the production of free radicals. These changes culminate in the cell aging process, predisposing to the emergence of neurodegenerative diseases such as Parkinson’s disease [52] and functional impairment in several systems, including the CNS [53].

Other group of proteins altered in the proteome was the heat shock proteins (HSP) family, found down-regulated in both exposed groups. In the 10 mg F/L group, there were chaperones such as HSP 70 1A (Q61696), 1B (P17879), 1-like (P16627), 4L (P48722), HSP 90 alpha (P07901), and beta (P11499), and in 50 mg F/L group, HSP 70 1-like (P16627), 1A (Q61696), and 1B (P17879), besides the HSP 90 alpha (P07901) and beta (P11499). These chaperones are responsible for folding, refolding, and protein remodeling, also acting in the transport and protection of these proteins. Still, in this context, the HSPs are also considered as markers of oxidative stress at the cellular level [54]. In view of this, we emphasize that the oxidative biochemistry imbalance in cerebellum of mice caused by exposure to fluoride was also able to modulate the proteomic profile.

Corroborating with the discussed above, we found the proteins superoxide dismutase (SOD) [Mn], mitochondrial (P09671), and superoxide dismutase [Cu-Zn] (P08228) down-regulated in exposed groups, showing an impact over this antioxidant component at a proteomic level. Several enzymatic and non-enzymatic mechanisms are associated with the antioxidant system, including the SOD, that are expressed as three distinct isoforms SOD1, SOD2, and SOD3. The SOD2 is in mitochondria, SOD3 is extracellular, and SOD1 is mainly found in cytoplasm [55]. Therefore, the literature shows that variations in this protein are related to motor diseases, such as amyotrophic lateral sclerosis [56], muscle weakness, and atrophy [57], mainly characterized by motor dysfunctions.

In our study, we found that exposure to NaF was able to promote biochemical and proteomic changes in the two exposure groups, however histological changes in Purkinje cells were not observed, which maintained their density in the cerebellar leaves. Within the cerebellar circuit, Purkinje cells perform the function of calibrating sensorimotor activities, in addition to giving plasticity of the cerebellar cortex to deep nuclei and directly contributing to the motor command [58,59]. We believe that the maintenance of the density of these cells in both exposed groups may be associated with the preservation of most of the motor functions evaluated in this investigation.

In this context, the cerebellar ability to perform compensatory activities against structural injuries in order to maintain the normal level of neurotransmission was kept [60,61]. In addition, other cerebellar populations, such as the granular, molecular layer, and deep cerebellar nuclei, participate in motor control and learning, helping to compensate for changes in synaptic structures [62,63]. These facts corroborate our analyses, suggesting that even in the face of observed biochemical and proteomic changes, neurochemical modulation may have occurred in order to maintain cerebellar homeostasis without, however, having cell death.

When investigating the possible functional repercussions that could be triggered by biochemical and molecular changes, we observed that fluoride promoted changes in the exploratory capacity, once the animals exposed to 50 mg F/L had the poorest performance on total a peripheral distances covered, as well as the animals exposed to 10 mg F/L had a shorter total distance covered. The number of rearings, i.e., the number of times the animal was in a vertical position, was significantly lower for both fluoride concentrations. In addition, during the assessment of postural stability of the animals using the inclined plane test, we observed changes in the strength of the hind limbs only of animals exposed to 50 mg F/L [64].

The performance of the pole test aimed to evaluate the muscle tone and bradykinesia of the animals. In this, the animal ability to rotate the body and descend to the platform was taken into account [65] and our results show that there was no change in these parameters after exposure to 10 mg F/L and 50 mg F/L.

The evaluation of coordination and motor refinement was performed using the rotarod. Damage to cerebellar structures compromises planning and execution during the same. We observed a greater number of falls in animals exposed to 50 mg F/L, demonstrating that there was impairment of motor activity in this experimental group. Thus, we observed that the motor changes promoted by exposure to fluoride were more evident in the group exposed to the highest dose. In addition, we reinforce that such motor tasks alterations were not related to differences in nutritional factors, since our fluoride challenge did not modify the body weight gain in the experimental groups.

In conclusion, with our in vivo model, it was possible to deepen the evidence related to the fluoride neurotoxicity. The unprecedent proteomic investigation combined with the oxidative biochemistry approach, provided several potential damage targets in cerebellum, providing evidence for further investigations. Moreover, we highlight that our in vivo model reinforces that long-term and low-level exposure to fluoride, at optimal concentrations for anticariogenic purposes, does not promote deleterious effects over cerebellar motor functions, while on the other hand, higher doses, equivalent to environmental pollution or water with natural high levels of fluoride, may pose serious public health concern, requiring clinical studies with humans and health care.

## 4. Materials and Methods

### 4.1. Experimental Animals and Exposure Protocol

This project was previously approved by the Ethics Committee on Experimental Animal Research of UFPA (CEPAE n° 2422071217). Thus, 60 male Swiss albino mice (21 days old) with body mass of approximately 10 g ± 5 g were placed in cages with 5 animals each, fed with balanced feed and water within an air-conditioned room with a light/dark cycle of 12 h light.

**Figure 9:**
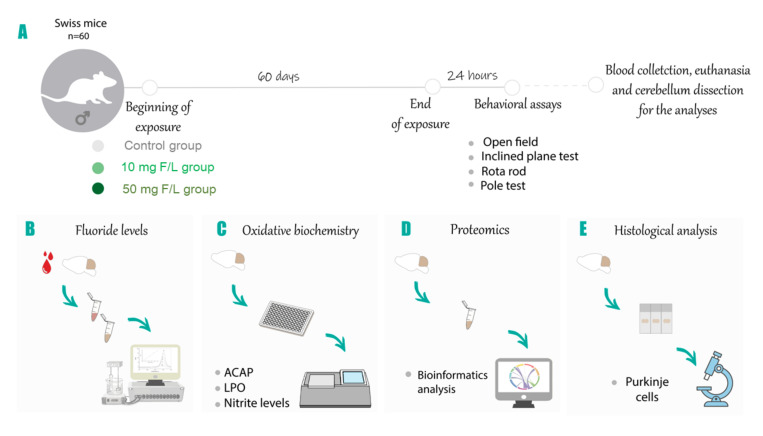
Description of samples and experimental steps. (**A**) Samples description and group division for 10 mg F/L, 50 mg F/L, or ultrapure water (H_2_O). After 24 h of the end of the exposure period behavioral evaluations were performed with the open field, inclined plane, rotarod, and pole test. (**B**) Analysis of the levels of fluoride present in the cerebellum; (**C**) analyses of oxidative biochemistry through the evaluation of the antioxidant capacity against peroxyl radicals (ACAP), lipid peroxidation (LPO), and nitrite levels; (**D**) characterization of the proteomic profile; (**E**) histological analysis through the quantification of Purkinje cells.

The animals were randomly divided into three groups (20 animals per group): 0 mg/L (control), 10 mg/L, and 50 mg/L of fluoride, as sodium fluoride (NaF). These concentrations are representative of the human exposure to artificially fluoridated water naturally fluoridated water from areas of endemic fluorosis, respectively, considering that rodents metabolize fluoride 5–10 times faster than humans [27,66]. The animals were exposed to the solutions through the bottle for a period of 60 days and the NaF was solubilized in ultrapure water as vehicle. The experimental design and methods used are summarized in the methodological Figure (Figure 9).

### 4.2. Behavioral Assessment

At the end of the exposure period, the animals were acclimatized for one hour in the assay room with sound attenuation and lighting intensity control, and subsequently, the animals were submitted to the battery of behavioral tests.

#### 4.2.1. Open Field

For the spontaneous locomotion analysis, the open field apparatus, webcam, computer, and timer were used, following the protocol as previously described [59,67]. Animals were placed on the arena (100 cm × 100 cm × 40 cm), wherein the floor is divided virtually into 25 equal quadrants (20 cm × 20 cm). Firstly, each animal was placed in the center of the floor and observed for five minutes. The number of total intersections was analyzed by the ANY-maze™ software (Stoelting Co., Wood Dale, IL, USA), and the number of standing position (rearing) was analyzed manually. To avoid motor memory process and consequently reduced motor exploitation, the animals were not submitted to habituation stage in this protocol [68,69,70,71].

#### 4.2.2. Inclined Plane

In this test, the animal’s ability to maintain postural stability was evaluated [70,71,72]. Animals with impaired motor coordination and equilibrium are unable to perform descent and ascent movements on a bar with a slope greater than 45°. According to the protocol of Teixeira et al. [73], the animals were submitted to a horizontal flat platform (Insight, São Paulo, Brazil), in which the angle of inclination was increased by 5° until the animal was able to maintain its position for up to 5 s. The latency until the fall and the final angle supported by the animal were counted at five consecutive trials (inter-trial interval of 60 s). The final score was resulted of the average angle calculated. This method has been validated by our group elsewhere [70,73].

#### 4.2.3. Pole Test

The Pole Test, initially described by Ogawa et al. [74], is an experiment used to evaluate movement disorders, in particular Bradykinesia [72,75]. The experiment was performed after initial acclimatization of 300 s (habituation phase) and the scores recorded over 5 trials, limited to 120 s each, with an interval of 60 s between them. The time needed for the animals to perform the task correctly was timed. Animals that failed to turn, in other words, slid across the beam in the same position as they were placed on the equipment, or fell, even if they had already turned upside down, were given the maximum time (120 s). The average was calculated with the 3 best times of each animal [72,76].

#### 4.2.4. Rotarod

The Rotarod (Insight^®^ Scientific Equipments, São Paulo, Brazil) consists of a grooved metal roller that has the ability to increase shaft rotations. The animals were trained to stay on the rotatory axis for a period of 3 min at 15 rotations per minute (habituation stage). After the training, the animals were submitted to the test, in which the time of the animal’s maintenance on the rotatory axis was counted in three test sessions of three minutes each [73,77].

### 4.3. Fluoride Levels Analysis

After the end of the behavioral tests, the animals were anesthetized and euthanized for the cerebellum collection, that were used for the quantification of fluoride levels, analyses of the oxidative biochemistry, and the proteomic profile. Ten animals per group had their cerebellum removed for fluoride ion analysis. Before the fluoride analysis was performed, plasma CO_2_ and tissue were removed by the adding of heated hexamethyldisiloxane (HMDS). Fluoride concentrations in the samples were determined in duplicate after diffusion facilitated by HMDS overnight [29,78], using the ion specific electrode (Orion Research, Model 9409, Espoo, Finland) and a miniature calomel electrode (Accumet # 13-620-79), both coupled to a potentiometer (Orion Research, Model EA 940).

Fluoride standards (0.0048 to 0.19 μg F) were prepared in triplicate and diffused in the same way as the samples. In addition, non-diffused standards were prepared to have exactly the same fluoride concentrations as the diffused standards. The millivoltage readings (mV) were converted to μg F using Excel (Microsoft). A standard curve with a correlation coefficient of r ≥ 0.99 was adopted. The comparison of mV readings shows that the fluoride in the diffused standards was completely extracted and analyzed (recovery > 95%).

### 4.4. Oxidative Biochemitry Analysis

#### 4.4.1. Antioxidant Capacity against Peroxyl Radicals (ACAP)

The evaluation of the antioxidant capacity against peroxyl radicals is through the determination of reactive oxygen species (ROS) and was performed according to several studies [79,80]. For the determination of ACAP, 2,2′ -azobis (2 methylpropionamidine) dihydrochloride (Aldrich ABAP; 4 mM) was added to the samples, which produces peroxyl radicals by thermal decomposition. After the first reading, the compound 2′,7′-cyclofluorescein diacetate (H2DCF-DA, Invitrogen, Carlsbad, CA, USA) at a concentration of 40 mM was added to the samples and readings were taken at an interval of 5 min for 1 h. The readings were measured with the aid of a fluorescence microplate reader (Victor X3, Perkin Elmer, Waltham, MA, USA). The results were calculated as the UF area difference x min in the same sample with and without ABAP addition and standardized to the ROS area without ABAP (background area). Using this methodology, a reduced relative area means higher antioxidant capacity, because low levels of fluorescence obtained after the addition of ABAP indicate high competence in neutralizing peroxyl radicals. For a direct reading of the results, the inverse of the relative difference between the ROS area with and without ABAP was considered as a measure of the antioxidant capacity. The results were expressed as percentage (%) of control.

#### 4.4.2. Determination of Lipid Peroxidation (LPO)

The determination of the LPO was based on our previous studies [60,80]. A solution containing methanesulfonic acid and indole N-methylphenyl (10.3 mM in acetonitrile) diluted in methanol (1:3) was added to the supernatant and incubated for 40 min at 45 °C. Absorbance was measured at 570 nm compared to standard malondialdehyde concentrations. The absorbance results were converted to the respective and corrected after measurement of the protein by the Bradford’s method [81].

#### 4.4.3. Determination of Nitrite Levels (NO)

For the determination of nitrite, we follow the protocol of agreement [11]. The samples were homogenized at 14,000 rpm. Only the supernatant of the samples was used for the analysis. The test to determine the level of nitrite was performed by the Griess method, which consists of the reaction of the Griess reagent (0.1% naphtilethylenediamine and 1% sulfanilamide in 5% phosphoric acid, 1:1, *v*/*v*) with 50 µL of the supernatant of each sample or 50 µL of standard sodium nitrite solution.

### 4.5. Characterization of the Proteomic Profile

#### 4.5.1. Protein Extraction

The proteomic analysis was performed as previously described by our group [11,82,83,84]. In the first step, a total of 10 animals per group were used and two samples were pooled, resulting in a final sample size of 5 per group. The samples were cryofructured using a cryogenic mill and liquid nitrogen followed by the extraction of proteins with lysis buffer (urea 7 M, thiourea 2 M, diluted in ammonium bicarbonate) in constant stirring at 4 °C, then the samples were centrifuged for 30 min at 14,000 rpm at 4 °C. The Bradford method was used for total protein quantification [81]. Subsequently, 50 µg proteins were diluted in 50 µL AMBIC (50 mM) and in each sample 10 µL AMBIC (50 mM) and 25 µL Rapigest (0.2%) (Waters Co., Manchester, UK) were added and incubated at 37 °C for 30 min. Subsequently, 2.5 µL of dithiothreitol (100 mM) was added and incubated at 37 °C for 60 min, then 2.5 µL iodoacetamide 300 mM (BioRad, Hercules, CA, USA) was added and incubated for 30 min at room temperature and in the dark. For the protein digestion process, 10 µL of trypsin (Thermo Fisher, Waltham, MA, USA) was added for 14 h at 37 °C, and in sequence 10 µL of 5% trifluoroacetic acid (Sigma-Aldrich, St. Louis, MO, USA) was added for 90 min at 37 °C and centrifuged at 14,000 rpm at 6 °C for 30 min. Subsequently, the supernatants were collected and purified using C18 rotary columns (Thermo Fisher, Waltham, MA, USA). The samples were resuspended in 12 µL of ADH (1 pmol·µL^−1^) + 108 µL of 3% acetonitrile (Sigma-Aldrich, St. Louis, MO, USA) and 0.1% formic acid (Thermo Fisher, Waltham, MA, USA).

#### 4.5.2. Mass Spectrometry

The identification and reading of the peptides in the samples was performed with nanoAcquity UPLCXevo QTof MS system (Waters, Mancester, UK), using the Protein Lynx Global Server (PLGS), as previously described by Lima Leite et al., (2014) [85]. The PLGS software, applying the Monte-Carlo algorithm, was used to obtain the protein expression difference between the groups, considering *p* < 0.05 for down-regulated proteins and *p* > 0.95 for up-regulated proteins. Protein identifications were determined by downloading Uniprot databases. Then, bioinformatic analyses were performed using Cytoscapes 3.6 (Java) with the ClueGO plug-in for the determination of biological process groups, based on Gene Ontology annotations [86].

### 4.6. Perfusion and Histological Processing

For the morphological analysis, 5 animals per group were randomized, anesthetized with a mixture of ketamine hydrochloride (90 mg/kg) and xylazine hydrochloride (9 mg/kg), and submitted to the perfusion process.

After perfusion, the cerebellums were removed from the cranial cap, post-fixed for 4 h in bouin solution and then processed in a battery of alcohol and xylol. Finally, they were included in Paraplast (McCormick, St. Louis, MO, USA.).

Coronal sections of 5 μm of the cerebellum thickness were obtained using a microtome and intended for tissue analysis. The previous preparation of the slides was performed with poly-D-lisin (Merck, Germany), in the sections that were assembled, right after the microtomy. Then, the sections were stained by hematoxylin for 10 min and washed in running water and then were immersed in eosin for 1 min. Subsequently, the slides were again washed in running water, dehydrated and diaphanized for mounting with Entellan (Merck Millipore, Burlington, MA, USA), and finally submitted to optical microscopy analysis.

#### Quantitative Analysis of Purkinje Neurons

The number of Purkinje cells was recorded using a Nikon Eclipse E200 binocular microscope (Tokyo, Japan), using a grid corresponding to an area of 0.0625 mm^2^ coupled to one of the eyepieces, in the 40× objective. Less than 3 fields in the cerebellum per section and 3 per animal in each group were analyzed, adapted from Lima et al. [87].

### 4.7. Statistical Analyses

To test the normality of the data, the Shapiro-Wilk test was performed. Then, the one-way ANOVA test was applied, followed by Tukey’s test to perform a comparison between the groups, considering the statistical significance level of *p* < 0.05. For the Rotarod test, the two-way ANOVA test was performed followed by Tukey’s test to perform comparison between the groups, considering the level of statistical significance of *p* < 0.05. The analyses of body weight gain were carried out using a two-way ANOVA repeated measure followed by Tukey’s post hoc test with *p* < 0.05. The data were expressed as mean ± standard error. GraphPad Prism 5.0 software (San Diego, CA, USA) was used for all analyses. In proteomic analyses, the PLGS software was used to obtain the difference of protein expression between the groups, applying the Monte-Carlo algorithm (*p* < 0.05 for down-regulated proteins and 1 − *p* > 0.95 for up-regulated proteins).

## 5. Conclusions

Within the doses investigated, fluoride promoted oxidative and proteomic alterations to the cerebellum, a primordial organ for motor control. Such changes did not result in motor deficits in the concentration regarded as therapeutical for caries control. However, for higher concentrations (related to those seen in endemic areas of dental fluorosis), significant alterations were observed. Studies in humans are needed to further investigate this relationship.

## Figures and Tables

**Table 1:** Biological processes distribution according to the altered proteins in the cerebellum of mice exposed to 10 mg F/L compared to control and 50 mg F/L compared to control. Proteins categorized based on Gene ontology, according to the ClueGo ^®^ plugin of the Cytoscape ^®^ 3.6 software. Significant terms (kappa score = 0.4) and distribution according to the percentage of the number of genes.

Groups	Biological Processes (% of Genes)
10 mg F/L vs. Control	Axon guidance (14.9%) Regulation of dendrite morphogenesis (9.6%) Positive regulation of DNA-binding transcription factor activity (8.8%) Regulation of axon extension (6.1%) Positive regulation of JNK cascade (4.4%) Regulation of alternative mRNA splicing, via spliceosome (4.4%) Acetyl-CoA biosynthetic process from pyruvate (3.5%) Activation of cysteine-type endopeptidase activity involved in apoptotic process (3.5%) Central nervous system neuron axogenesis (3.5%) Cysteine-type endopeptidase inhibitor activity involved in apoptotic process (3.5%) Hypothalamus gonadotrophin-releasing hormone neuron development (3.5%) mRNA splice site selection (3.5%) negative regulation of mRNA splicing, via spliceosome (3.5%) Regulation of calcium ion transport into cytosol (3.5%) Dendrite extension (2.6%) Glycolytic process through fructose-6-phosphate (2.6%) Ionotropic glutamate receptor activity (2.6%) Mitochondrial ATP synthesis coupled proton transport (2.6%) Nuclear receptor activity (2.6%) Positive regulation of ATP biosynthetic process (2.6%) Positive regulation of sprouting angiogenesis (2.6%) Potassium: proton exchanging ATPase activity (2.6%) Regulation of glycolytic process (2.6%)
50 mg F/L vs. Control	Axon guidance (13.6%) Regulation of axogenesis (13.6%) Regulation of release of sequestered calcium ion into cytosol (9.1%) Dendritic spine morphogenesis (7.3%) Activation of cysteine-type endopeptidase activity involved in apoptotic process (4.5%) Regulation of alternative mRNA splicing, via spliceosome (4.5%) Cysteine-type endopeptidase inhibitor activity involved in apoptotic process (4.5%) Dendrite extension (4.5%) Negative regulation of mRNA splicing, via spliceosome (4.5%) mRNA splice site selection (3.6%) Barbed-end actin filament capping (3.6%) Central nervous system neuron axogenesis (3.6%) Mitochondrial ATP synthesis coupled proton transport (3.6%) Pyruvate dehydrogenase (NAD+) activity (3.6%) Ionotropic glutamate receptor activity (3.6%) Canonical glycolysis (3.6%) Auditory receptor cell morphogenesis (2.7%) Regulation of glycolytic process (2.7%) Potassium: proton exchanging ATPase activity (2.7%)

## Supplementary Materials

Supplementary Materials can be found at https://www.mdpi.com/1422-0067/21/19/7297/s1.

## Abbreviations

NaF	Sodium Fluoride
ACAP	Antioxidant Capacity Against Peroxyl Radicals
MDA	Lipid Peroxidation
NO	Nitrite
CNS	Central Nervous System
HF	Hydrogen Fluoride
MAPK	Mitogen-Activated Protein Kinases
AP 1	Activator Protein 1
NF-B	Nuclear Factor Kappa B
ROS	Reactive Oxygen Species
HSP	Heat Shock Proteins
SOD	Superoxide Dismutase
