# Supplementary material for: Effects of Fluoride Long-Term Exposure over the Cerebellum: Global Proteomic Profile, Oxidative Biochemistry, Cell Density, and Motor Behavior Evaluation
Source: Int J Mol Sci. 2020 Oct 2;21(19):7297. doi: 10.3390/ijms21197297 (PMC7582550; doi:10.3390/ijms21197297)
Supplement: Supplementary file 1 [file ijms-21-07297-s001.pdf]

Table S1. Identified proteins with exclusive expression in cerebellum of rats of control, 10mg F/L and 50mg F/L groups.

Accession ID[a]	Protein Name	*PLGS* Score	Group
Q3TXS7	26S proteasome non-ATPase regulatory subunit 1	435	Control
Q9CQX8	28S ribosomal protein S36_ mitochondrial	197	Control
P52760	2-iminobutanoate/2-iminopropanoate deaminase	315	Control
Q60597	2-oxoglutarate dehydrogenase_ mitochondrial	67	Control
P24815	3 beta-hydroxysteroid dehydrogenase/Delta 5-->4-isomerase type 1	84	Control
Q99L13	3-hydroxyisobutyrate dehydrogenase_ mitochondrial	114	Control
P61922	4-aminobutyrate aminotransferase_ mitochondrial	470	Control
P10852	4F2 cell-surface antigen heavy chain	220	Control
Q8K010	5-oxoprolinase	197	Control
P47955	60S acidic ribosomal protein P1	190	Control
P70266	6-phosphofructo-2-kinase/fructose-2_6-bisphosphatase 1	113	Control
Q8QZT1	Acetyl-CoA acetyltransferase_ mitochondrial	402	Control
Q9R0Y5	Adenylate kinase isoenzyme 1	623	Control
Q80TS3	Adhesion G protein-coupled receptor L3	59	Control
B7ZCC9	Adhesion G-protein coupled receptor G4	139	Control
Q6P5E6	ADP-ribosylation factor-binding protein GGA2	45	Control
E9Q394	A-kinase anchor protein 13	60	Control
Q80Y20	Alkylated DNA repair protein alkB homolog 8	111	Control
P07758	Alpha-1-antitrypsin 1-1	78	Control
P22599	Alpha-1-antitrypsin 1-2	78	Control
Q00896	Alpha-1-antitrypsin 1-3	78	Control
Q00897	Alpha-1-antitrypsin 1-4	78	Control
P57780	Alpha-actinin-4	58	Control
Q9QYC0	Alpha-adducin	270	Control
Q9DB05	Alpha-soluble NSF attachment protein	156	Control

Q6PAM1	Alpha-taxilin	161	Control
Q7TQF7	Amphiphysin	62	Control
Q9DBR4	Amyloid-beta A4 precursor protein-binding family B member 2	152	Control
Q3V0J4	Ankyrin repeat domain-containing protein 53	76	Control
Q8C8R3	Ankyrin-2	46	Control
P14824	Annexin A6	107	Control
O35643	AP-1 complex subunit beta-1	105	Control
P17426	AP-2 complex subunit alpha-1	279	Control
P17427	AP-2 complex subunit alpha-2	318	Control
Q9DBG3	AP-2 complex subunit beta	91	Control
P84091	AP-2 complex subunit mu	181	Control
Q5YD48	APOBEC1 complementation factor	72	Control
E9Q414	Apolipoprotein B-100	95	Control
P03930	ATP synthase protein 8	864	Control
Q9D3D9	ATP synthase subunit delta_ mitochondrial	1133	Control
Q06185	ATP synthase subunit e_ mitochondrial	268	Control
Q91VR2	ATP synthase subunit gamma_ mitochondrial	248	Control
P97450	ATP synthase-coupling factor 6_ mitochondrial	987	Control
Q3UMC0	ATPase family protein 2 homolog	324	Control
Q9DC29	ATP-binding cassette sub-family B member 6_ mitochondrial	61	Control
Q6P542	ATP-binding cassette sub-family F member 1	43	Control
Q14BI7	ATP-dependent RNA helicase TDRD9	106	Control
Q9WV92	Band 4.1-like protein 3	243	Control
P59017	Bcl-2-like protein 13	75	Control
Q99L88	Beta-1-syntrophin	72	Control
Q9QYB8	Beta-adducin	129	Control
P28663	Beta-soluble NSF attachment protein	394	Control
Q6WVG3	BTB/POZ domain-containing protein KCTD12	193	Control
P11798	Calcium/calmodulin-dependent protein kinase type II subunit alpha	685	Control
P28652	Calcium/calmodulin-dependent protein kinase type II subunit beta	873	Control

Q6PHZ2	Calcium/calmodulin-dependent protein kinase type II subunit delta	685	Control
Q9QXX4	Calcium-binding mitochondrial carrier protein Aralar2	74	Control
Q8BYR5	Calcium-dependent secretion activator 2	76	Control
Q08331	Calretinin	422	Control
P68181	cAMP-dependent protein kinase catalytic subunit beta	124	Control
P00920	Carbonic anhydrase 2	420	Control
O54833	Casein kinase II subunit alpha'	81	Control
P18242	Cathepsin D	345	Control
Q99N28	Cell adhesion molecule 3	168	Control
A2AL36	Centriolin	138	Control
Q9CZU6	Citrate synthase_ mitochondrial	759	Control
Q6IRU5	Clathrin light chain B	78	Control
P54751	CMP-N-acetylneuraminate-beta-galactosamide-alpha-2_3-sialyltransferase 1	95	Control
Q8CFW7	Coiled-coil and C2 domain-containing protein 2A	47	Control
O35206	Collagen alpha-1(XV) chain	106	Control
P12960	Contactin-1	138	Control
Q6P8J7	Creatine kinase S-type_ mitochondrial	43	Control
P49919	Cyclin-dependent kinase inhibitor 1C	144	Control
Q9Z1J3	Cysteine desulfurase_ mitochondrial	173	Control
P99028	Cytochrome b-c1 complex subunit 6_ mitochondrial	251	Control
Q9D855	Cytochrome b-c1 complex subunit 7	334	Control
P19536	Cytochrome c oxidase subunit 5B_ mitochondrial	122	Control
P43024	Cytochrome c oxidase subunit 6A1_ mitochondrial	1081	Control
P56391	Cytochrome c oxidase subunit 6B1	615	Control
P48771	Cytochrome c oxidase subunit 7A2_ mitochondrial	531	Control
Q62425	Cytochrome c oxidase subunit NDUFA4	1401	Control
P62897	Cytochrome c_ somatic	336	Control
Q9D0M3	Cytochrome c1_ heme protein_ mitochondrial	1841	Control
Q91V12	Cytosolic acyl coenzyme A thioester hydrolase	243	Control
Q80TR8	DDB1- and CUL4-associated factor 1	65	Control

Q3TUL7	DDB1- and CUL4-associated factor 17	69	Control
Q99KU1	Dehydrodolichyl diphosphate synthase complex subunit Dhdds	174	Control
Q9Z110	Delta-1-pyrroline-5-carboxylate synthase	112	Control
Q9WV69	Dematin	128	Control
Q8BTT6	Digestive organ expansion factor homolog	150	Control
O08749	Dihydrolipoyl dehydrogenase_ mitochondrial	352	Control
Q8BMF4	Dihydrolipoyllysine-residue acetyltransferase component of pyruvate dehydrogenase complex_ mitochondrial	286	Control
Q9D2G2	Dihydrolipoyllysine-residue succinyltransferase component of 2-oxoglutarate dehydrogenase complex_ mitochondrial	76	Control
Q8BVI4	Dihydropteridine reductase	552	Control
Q811D0	Disks large homolog 1	75	Control
Q91XM9	Disks large homolog 2	162	Control
Q62108	Disks large homolog 4	75	Control
Q9CWV1	DNA helicase MCM8	69	Control
Q8C7R7	DNA-binding protein RFX6	57	Control
Q5U458	DnaJ homolog subfamily C member 11	181	Control
P70169	Double C2-like domain-containing protein beta	58	Control
Q9D3V1	Dynein regulatory complex protein 10	65	Control
O88343	Electrogenic sodium bicarbonate cotransporter 1	87	Control
Q99LC5	Electron transfer flavoprotein subunit alpha_ mitochondrial	242	Control
Q99MI1	ELKS/Rab6-interacting/CAST family member 1	65	Control
Q8BFR5	Elongation factor Tu_ mitochondrial	209	Control
Q3TGW2	Endonuclease/exonuclease/phosphatase family domain-containing protein 1	92	Control
Q62420	Endophilin-A1	1559	Control
Q62419	Endophilin-A2	1160	Control
P08113	Endoplasmin	57	Control
Q9DCS3	Enoyl-[acyl-carrier-protein] reductase_ mitochondrial	155	Control
P54754	Ephrin type-B receptor 3	87	Control
P60843	Eukaryotic initiation factor 4A-I	357	Control

P10630	Eukaryotic initiation factor 4A-II	312	Control
Q91VC3	Eukaryotic initiation factor 4A-III	241	Control
P43006	Excitatory amino acid transporter 2	258	Control
Q924C1	Exportin-5	74	Control
P47754	F-actin-capping protein subunit alpha-2	146	Control
Q8BHL6	Fanconi anemia core complex-associated protein 24	205	Control
P51880	Fatty acid-binding protein_ brain	262	Control
A2A6H3	F-box only protein 47	51	Control
Q9QXE7	F-box-like/WD repeat-containing protein TBL1X	59	Control
Q8CI03	FLYWCH-type zinc finger-containing protein 1	151	Control
P97807	Fumarate hydratase_ mitochondrial	398	Control
P23242	Gap junction alpha-1 protein	229	Control
Q9JI57	General transcription factor II-I repeat domain-containing protein 1	138	Control
P03995	Glial fibrillary acidic protein	177	Control
P06745	Glucose-6-phosphate isomerase	2193	Control
Q61625	Glutamate receptor ionotropic_ delta-2	85	Control
D3Z7P3	Glutaminase kidney isoform_ mitochondrial	115	Control
P19639	Glutathione S-transferase Mu 3	173	Control
P48774	Glutathione S-transferase Mu 5	115	Control
O35660	Glutathione S-transferase Mu 6	223	Control
P19157	Glutathione S-transferase P 1	328	Control
P46425	Glutathione S-transferase P 2	157	Control
Q64521	Glycerol-3-phosphate dehydrogenase_ mitochondrial	100	Control
Q8CI94	Glycogen phosphorylase_ brain form	194	Control
Q9ET01	Glycogen phosphorylase_ liver form	82	Control
Q9WUB3	Glycogen phosphorylase_ muscle form	110	Control
Q9CW79	Golgin subfamily A member 1	136	Control
Q3V0G7	GTPase-activating Rap/Ran-GAP domain-like protein 3	152	Control
P62827	GTP-binding nuclear protein Ran	253	Control
P21279	Guanine nucleotide-binding protein G(q) subunit alpha	238	Control

P21278	Guanine nucleotide-binding protein subunit alpha-11	61	Control
Q8CFB4	Guanylate-binding protein 5	58	Control
P48722	Heat shock 70 kDa protein 4L	541	Control
P22361	Hepatocyte nuclear factor 1-alpha	66	Control
Q60668	Heterogeneous nuclear ribonucleoprotein D0	103	Control
P61979	Heterogeneous nuclear ribonucleoprotein K	1539	Control
Q8R081	Heterogeneous nuclear ribonucleoprotein L	125	Control
Q8VEK3	Heterogeneous nuclear ribonucleoprotein U	163	Control
O88569	Heterogeneous nuclear ribonucleoproteins A2/B1	341	Control
P17710	Hexokinase-1	327	Control
O08528	Hexokinase-2	190	Control
P70349	Histidine triad nucleotide-binding protein 1	1124	Control
A2A935	Histone-lysine N-methyltransferase PRDM16	254	Control
Q80W88	Homeobox and leucine zipper protein Homez	191	Control
P31310	Homeobox protein Hox-A10	70	Control
Q3UNN8	Inactive phospholipase D5	69	Control
Q920Q8	Influenza virus NS1A-binding protein homolog	258	Control
P11881	Inositol 1_4_5-trisphosphate receptor type 1	398	Control
A2ARP1	Inositol hexakisphosphate and diphosphoinositol-pentakisphosphate kinase 1	108	Control
Q80SU7	Interferon-induced very large GTPase 1	57	Control
O89109	Intermediate conductance calcium-activated potassium channel protein 4	103	Control
Q9QY61	Iroquois-class homeodomain protein IRX-4	58	Control
P85094	Isochorismatase domain-containing protein 2A	212	Control
Q9QYN3	Kallikrein-11	128	Control
Q9D3R6	Katanin p60 ATPase-containing subunit A-like 2	234	Control
Q61595	Kinectin	273	Control
B1AVY7	Kinesin-like protein KIF16B	31	Control
P33173	Kinesin-like protein KIF1A	268	Control
Q60575	Kinesin-like protein KIF1B	17	Control
Q9QXL1	Kinesin-like protein KIF21B	56	Control

Q8CFA7	Krueppel-like factor 17	162	Control
Q9CPU0	Lactoylglutathione lyase	239	Control
P02468	Laminin subunit gamma-1	331	Control
Q9JIA1	Leucine-rich glioma-inactivated protein 1	49	Control
O88520	Leucine-rich repeat protein SHOC-2	106	Control
Q52KR2	Leucine-rich repeats and immunoglobulin-like domains protein 2	54	Control
Q8BM14	Lipase member K	112	Control
O35711	Liprin-beta-2	116	Control
Q3UZ18	Little elongation complex subunit 2	308	Control
P06151	L-lactate dehydrogenase A chain	1657	Control
P00342	L-lactate dehydrogenase C chain	1423	Control
Q91WC3	Long-chain-fatty-acid--CoA ligase 6	158	Control
Q6P1G2	Lysine-specific demethylase 2B	117	Control
P60755	MAM domain-containing glycosylphosphatidylinositol anchor protein 2	53	Control
P05532	Mast/stem cell growth factor receptor Kit	64	Control
Q8K310	Matrin-3	79	Control
Q9D2X5	MAU2 chromatid cohesion factor homolog	95	Control
Q8BQM9	Mediator of RNA polymerase II transcription subunit 12-like protein	102	Control
Q3UHE1	Membrane-associated phosphatidylinositol transfer protein 3	89	Control
Q8C1A3	Methionine synthase reductase	132	Control
Q9JLZ3	Methylglutaconyl-CoA hydratase_ mitochondrial	585	Control
Q9EQ20	Methylmalonate-semialdehyde dehydrogenase [acylating]_ mitochondrial	183	Control
Q8CAQ8	MICOS complex subunit Mic60	247	Control
P20357	Microtubule-associated protein 2	88	Control
Q9R1L5	Microtubule-associated serine/threonine-protein kinase 1	122	Control
Q60592	Microtubule-associated serine/threonine-protein kinase 2	112	Control
Q3U214	Microtubule-associated serine/threonine-protein kinase 3	112	Control
Q811L6	Microtubule-associated serine/threonine-protein kinase 4	144	Control
Q9CW42	Mitochondrial amidoxime-reducing component 1	167	Control
P63085	Mitogen-activated protein kinase 1	123	Control

Q80Y86	Mitogen-activated protein kinase 15	56	Control
Q63844	Mitogen-activated protein kinase 3	64	Control
Q6P5G0	Mitogen-activated protein kinase 4	57	Control
P20917	Myelin-associated glycoprotein	250	Control
Q61885	Myelin-oligodendrocyte glycoprotein	381	Control
Q69ZQ1	Myogenesis-regulating glycosidase	115	Control
Q80YT7	Myomegalin	55	Control
Q5DTJ9	Myopalladin	210	Control
Q61879	Myosin-10	125	Control
Q8VDD5	Myosin-9	58	Control
Q99LD8	N(G)_N(G)-dimethylarginine dimethylaminohydrolase 2	283	Control
Q6P5U7	NACHT and WD repeat domain-containing protein 2	46	Control
Q8VDQ8	NAD-dependent protein deacetylase sirtuin-2	96	Control
Q99LC3	NADH dehydrogenase [ubiquinone] 1 alpha subcomplex subunit 10_ mitochondrial	455	Control
Q9ERS2	NADH dehydrogenase [ubiquinone] 1 alpha subcomplex subunit 13	444	Control
Q9CQ91	NADH dehydrogenase [ubiquinone] 1 alpha subcomplex subunit 3	580	Control
Q9DCJ5	NADH dehydrogenase [ubiquinone] 1 alpha subcomplex subunit 8	300	Control
Q9DCS9	NADH dehydrogenase [ubiquinone] 1 beta subcomplex subunit 10	114	Control
Q91YT0	NADH dehydrogenase [ubiquinone] flavoprotein 1_ mitochondrial	578	Control
Q9D6J6	NADH dehydrogenase [ubiquinone] flavoprotein 2_ mitochondrial	766	Control
Q91WD5	NADH dehydrogenase [ubiquinone] iron-sulfur protein 2_ mitochondrial	221	Control
Q9DCT2	NADH dehydrogenase [ubiquinone] iron-sulfur protein 3_ mitochondrial	247	Control
P52503	NADH dehydrogenase [ubiquinone] iron-sulfur protein 6_ mitochondrial	380	Control
Q8K3J1	NADH dehydrogenase [ubiquinone] iron-sulfur protein 8_ mitochondrial	177	Control
Q91VD9	NADH-ubiquinone oxidoreductase 75 kDa subunit_ mitochondrial	264	Control
P13595	Neural cell adhesion molecule 1	138	Control
P06837	Neuromodulin	194	Control
O70174	Neuronal acetylcholine receptor subunit alpha-4	113	Control
P35803	Neuronal membrane glycoprotein M6-b	316	Control

Q8BVW0	Neutral alpha-glucosidase C	49	Control
E9Q7G0	Nuclear mitotic apparatus protein 1	147	Control
Q60974	Nuclear receptor corepressor 1	153	Control
Q3UHX0	Nucleolar protein 8	110	Control
Q61937	Nucleophosmin	271	Control
P59235	Nucleoporin Nup43	70	Control
P15532	Nucleoside diphosphate kinase A	1453	Control
Q01768	Nucleoside diphosphate kinase B	1383	Control
Q9EPK6	Nucleotide exchange factor SIL1	37	Control
O88708	Origin recognition complex subunit 4	84	Control
Q3URU2	Paternally-expressed gene 3 protein	128	Control
P70271	PDZ and LIM domain protein 4	71	Control
Q9Z0G0	PDZ domain-containing protein GIPC1	136	Control
Q6RUU0	Pentraxin-4	93	Control
P26883	Peptidyl-prolyl cis-trans isomerase FKBP1A	1877	Control
P15331	Peripherin	44	Control
Q61171	Peroxiredoxin-2	1174	Control
O08807	Peroxiredoxin-4	215	Control
Q8K0S0	Phytanoyl-CoA hydroxylase-interacting protein	171	Control
Q8BGT8	Phytanoyl-CoA hydroxylase-interacting protein-like	78	Control
G5E829	Plasma membrane calcium-transporting ATPase 1	198	Control
Q9R0K7	Plasma membrane calcium-transporting ATPase 2	90	Control
P26618	Platelet-derived growth factor receptor alpha	178	Control
P05622	Platelet-derived growth factor receptor beta	71	Control
E9QPE2	Platelet-derived growth factor receptor beta	71	Control
Q80TI1	Pleckstrin homology domain-containing family H member 1	541	Control
P60335	Poly(rC)-binding protein 1	243	Control
Q61990	Poly(rC)-binding protein 2	311	Control
P57722	Poly(rC)-binding protein 3	140	Control
Q91Z31	Polypyrimidine tract-binding protein 2	179	Control

Q8BHJ9	Pre-mRNA-splicing factor SLU7	283	Control
A2AKX3	Probable helicase senataxin	74	Control
P62962	Profilin-1	325	Control
Q91ZA3	Propionyl-CoA carboxylase alpha chain_ mitochondrial	174	Control
Q6PDI5	Proteasome adapter and scaffold protein ECM29	155	Control
Q99JB7	Protein amnionless	151	Control
Q924A2	Protein capicua homolog	53	Control
P27773	Protein disulfide-isomerase A3	82	Control
E9Q8I9	Protein furry homolog	88	Control
P20444	Protein kinase C alpha type	52	Control
Q61644	Protein kinase C and casein kinase substrate in neurons protein 1	133	Control
P68404	Protein kinase C beta type	61	Control
P63318	Protein kinase C gamma type	138	Control
Q9QYG0	Protein NDRG2	586	Control
O55125	Protein NipSnap homolog 1	430	Control
Q9D7Y9	Protein SLX4IP	409	Control
Q9QX47	Protein SON	75	Control
Q68FL4	Putative adenosylhomocysteinase 3	390	Control
Q9D8C3	Putative bifunctional UDP-N-acetylglucosamine transferase and deubiquitinase ALG13	150	Control
Q91W97	Putative hexokinase HKDC1	165	Control
Q8K183	Pyridoxal kinase	79	Control
Q05920	Pyruvate carboxylase_ mitochondrial	161	Control
P35486	Pyruvate dehydrogenase E1 component subunit alpha_ somatic form_ mitochondrial	129	Control
P63001	Ras-related C3 botulinum toxin substrate 1	636	Control
Q05144	Ras-related C3 botulinum toxin substrate 2	486	Control
P60764	Ras-related C3 botulinum toxin substrate 3	486	Control
Q9DD03	Ras-related protein Rab-13	484	Control
Q00342	Receptor-type tyrosine-protein kinase FLT3	64	Control

Q0VGM9	Regulator of telomere elongation helicase 1	137	Control
Q8K0T0	Reticulon-1	94	Control
Q6NS82	Reticulophagy regulator 2	74	Control
Q99PT1	Rho GDP-dissociation inhibitor 1	398	Control
Q8BL80	Rho GTPase-activating protein 22	222	Control
Q3UPH7	Rho guanine nucleotide exchange factor 40	146	Control
Q8K4I3	Rho guanine nucleotide exchange factor 6	181	Control
Q91VI7	Ribonuclease inhibitor	98	Control
Q8R1Z9	RING finger protein 121	147	Control
Q8CCI5	RING1 and YY1-binding protein	121	Control
Q7TT28	RNA exonuclease 1 homolog	109	Control
Q0VBL3	RNA-binding protein 15	58	Control
Q9CXK9	RNA-binding protein 33	62	Control
Q8JZV4	RNA-binding protein 41	84	Control
Q80SW1	S-adenosylhomocysteine hydrolase-like protein 1	390	Control
Q5U680	S-adenosylmethionine mitochondrial carrier protein	339	Control
O55143	Sarcoplasmic/endoplasmic reticulum calcium ATPase 2	62	Control
Q8K021	Secretory carrier-associated membrane protein 1	139	Control
Q8C650	Septin-10	310	Control
Q8C1B7	Septin-11	397	Control
Q9DA97	Septin-14	310	Control
P28661	Septin-4	88	Control
O55131	Septin-7	627	Control
Q8CHH9	Septin-8	419	Control
Q6PDM2	Serine/arginine-rich splicing factor 1	223	Control
Q99MW1	Serine/threonine-protein kinase 31	62	Control
P04627	Serine/threonine-protein kinase A-Raf	140	Control
Q7TT50	Serine/threonine-protein kinase MRCK beta	74	Control
O54949	Serine/threonine-protein kinase NLK	64	Control

Q76MZ3	Serine/threonine-protein phosphatase 2A 65 kDa regulatory subunit A alpha isoform	78	Control
P63328	Serine/threonine-protein phosphatase 2B catalytic subunit alpha isoform	221	Control
P48453	Serine/threonine-protein phosphatase 2B catalytic subunit beta isoform	141	Control
Q9CQV3	Serpin B11	132	Control
Q91WA6	Sharpin	60	Control
Q6S5L9	SHC-transforming protein 4	71	Control
Q62230	Sialoadhesin	86	Control
Q99JR1	Sideroflexin-1	106	Control
Q91V61	Sideroflexin-3	89	Control
Q9CYZ8	Single-stranded DNA-binding protein 2	89	Control
Q5XG71	Small subunit processome component 20 homolog	112	Control
Q9D3S3	Sorting nexin-29	54	Control
P15508	Spectrin beta chain_ erythrocytic	42	Control
Q5U4C3	Splicing factor_ arginine/serine-rich 19	73	Control
P38647	Stress-70 protein_ mitochondrial	102	Control
Q60864	Stress-induced-phosphoprotein 1	100	Control
Q8K2B3	Succinate dehydrogenase [ubiquinone] flavoprotein subunit_ mitochondrial	175	Control
Q9CQA3	Succinate dehydrogenase [ubiquinone] iron-sulfur subunit_ mitochondrial	216	Control
Q9Z2I9	Succinate--CoA ligase [ADP-forming] subunit beta_ mitochondrial	296	Control
Q8BWF0	Succinate-semialdehyde dehydrogenase_ mitochondrial	299	Control
Q9D0K2	Succinyl-CoA:3-ketoacid coenzyme A transferase 1_ mitochondrial	186	Control
P58735	Sulfate anion transporter 1	95	Control
P09671	Superoxide dismutase [Mn]_ mitochondrial	161	Control
P46096	Synaptotagmin-1	213	Control
P46097	Synaptotagmin-2	208	Control
P80314	T-complex protein 1 subunit beta	80	Control
Q8BYI9	Tenascin-R	60	Control
Q62288	Testican-1	327	Control
P20108	Thioredoxin-dependent peroxide reductase_ mitochondrial	227	Control

Q60610	T-lymphoma invasion and metastasis-inducing protein 1	79	Control
Q99MB1	Toll-like receptor 3	107	Control
Q5SRX1	TOM1-like protein 2	117	Control
P70399	TP53-binding protein 1	119	Control
Q8BV79	TPR and ankyrin repeat-containing protein 1	97	Control
Q93092	Transaldolase	87	Control
Q61286	Transcription factor 12	100	Control
Q62318	Transcription intermediary factor 1-beta	67	Control
P42669	Transcriptional activator protein Pur-alpha	178	Control
Q9R1Q8	Transgelin-3	276	Control
Q7TN37	Transient receptor potential cation channel subfamily M member 4	79	Control
Q01853	Transitional endoplasmic reticulum ATPase	278	Control
P40142	Transketolase	204	Control
Q80W04	Transmembrane and coiled-coil domains protein 2	77	Control
A2AAE1	Transmembrane protein KIAA1109	85	Control
Q8BMS1	Trifunctional enzyme subunit alpha_ mitochondrial	57	Control
A2ADA5	tRNA pseudouridine synthase-like 1	119	Control
P21107	Tropomyosin alpha-3 chain	119	Control
Q62270	Tyrosine-protein kinase Srms	84	Control
Q3UN04	Ubiquitin carboxyl-terminal hydrolase 30	73	Control
Q9R0P9	Ubiquitin carboxyl-terminal hydrolase isozyme L1	735	Control
Q7TQI3	Ubiquitin thioesterase OTUB1	531	Control
Q9D6A1	Unconventional myosin-Ih	121	Control
Q2QI47	Usherin	66	Control
Q8R164	Valacyclovir hydrolase	94	Control
P35969	Vascular endothelial growth factor receptor 1	64	Control
P35918	Vascular endothelial growth factor receptor 2	64	Control
P35917	Vascular endothelial growth factor receptor 3	84	Control
Q62059	Versican core protein	223	Control
P62761	Visinin-like protein 1	235	Control

Q8CC88	von Willebrand factor A domain-containing protein 8	68	Control
Q9Z1G4	V-type proton ATPase 116 kDa subunit a isoform 1	209	Control
P51863	V-type proton ATPase subunit d 1	263	Control
P50518	V-type proton ATPase subunit E 1	392	Control
Q8BVE3	V-type proton ATPase subunit H	215	Control
O88466	Zinc finger protein 106	151	Control
Q810A1	Zinc finger protein 18	57	Control
Q61464	Zinc finger protein 638	164	Control
Q9Z1B3	1-phosphatidylinositol 4_5-bisphosphate phosphodiesterase beta-1	41	10PPM
A2AP18	1-phosphatidylinositol 4_5-bisphosphate phosphodiesterase eta-2	25	10PPM
P84078	ADP-ribosylation factor 1	1615	10PPM
Q8BSL7	ADP-ribosylation factor 2	1135	10PPM
P61205	ADP-ribosylation factor 3	1615	10PPM
P61750	ADP-ribosylation factor 4	815	10PPM
P84084	ADP-ribosylation factor 5	815	10PPM
P61164	Alpha-centractin	397	10PPM
Q8BZQ7	Anaphase-promoting complex subunit 2	88	10PPM
Q8C6Y6	Ankyrin repeat and SOCS box protein 14	58	10PPM
G5E8K5	Ankyrin-3	246	10PPM
P06728	Apolipoprotein A-IV	181	10PPM
Q99KN1	Arrestin domain-containing protein 1	175	10PPM
Q99PU8	ATP-dependent RNA helicase DHX30	149	10PPM
Q80XK6	Autophagy-related protein 2 homolog B	57	10PPM
Q9Z2H5	Band 4.1-like protein 1	55	10PPM
Q8R5C5	Beta-centractin	409	10PPM
Q8VBT1	Beta-taxilin	41	10PPM
Q91XV3	Brain acid soluble protein 1	334	10PPM
Q8BKX1	Brain-specific angiogenesis inhibitor 1-associated protein 2	131	10PPM
Q61361	Brevican core protein	69	10PPM
Q9D6P8	Calmodulin-like protein 3	1622	10PPM

Q9R1S8	Calpain-7	68	10PPM
Q65CL1	Catenin alpha-3	54	10PPM
P35762	CD81 antigen	628	10PPM
A2A6Q5	Cell division cycle protein 27 homolog	60	10PPM
Q6A065	Centrosomal protein of 170 kDa	88	10PPM
A2A8L1	Chromodomain-helicase-DNA-binding protein 5	41	10PPM
P49025	Citron Rho-interacting kinase	25	10PPM
Q9EPU4	Cleavage and polyadenylation specificity factor subunit 1	138	10PPM
Q8CDI7	Coiled-coil domain-containing protein 150	74	10PPM
Q8CDV0	Coiled-coil domain-containing protein 178	29	10PPM
Q8C4S8	DENN domain-containing protein 2A	65	10PPM
Q9EQF6	Dihydropyrimidinase-related protein 5	71	10PPM
Q6PFD5	Disks large-associated protein 3	52	10PPM
Q3UYV8	Dynein assembly factor 3_ axonemal	79	10PPM
Q4U2R1	E3 ubiquitin-protein ligase HERC2	88	10PPM
Q0VAV2	Exophilin-5	38	10PPM
Q61553	Fascin	96	10PPM
Q80X90	Filamin-B	32	10PPM
Q8BML9	Glutamine--tRNA ligase	95	10PPM
Q61316	Heat shock 70 kDa protein 4	103	10PPM
Q61699	Heat shock protein 105 kDa	59	10PPM
Q9WV07	Hydroperoxide isomerase ALOXE3	59	10PPM
Q3KNY0	Immunoglobulin-like and fibronectin type III domain-containing protein 1	30	10PPM
Q63ZW7	InaD-like protein	36	10PPM
Q80V86	Integrator complex subunit 8	71	10PPM
A6X935	Inter alpha-trypsin inhibitor_ heavy chain 4	43	10PPM
Q60625	Intercellular adhesion molecule 5	49	10PPM
Q9D8C4	Interferon-induced 35 kDa protein homolog	307	10PPM
Q62406	Interleukin-1 receptor-associated kinase 1	143	10PPM
A2CG49	Kalirin	83	10PPM

P11369	LINE-1 retrotransposable element ORF2 protein	40	10PPM
Q9JI18	Low-density lipoprotein receptor-related protein 1B	39	10PPM
Q9JJ78	Lymphokine-activated killer T-cell-originated protein kinase	70	10PPM
Q61847	Meprin A subunit beta	90	10PPM
B1AYB6	Methyl-CpG-binding domain protein 5	85	10PPM
Q9QYR6	Microtubule-associated protein 1A	43	10PPM
P10637	Microtubule-associated protein tau	156	10PPM
Q9D6M3	Mitochondrial glutamate carrier 1	1914	10PPM
Q9DB41	Mitochondrial glutamate carrier 2	265	10PPM
Q9CWS0	N(G)_N(G)-dimethylarginine dimethylaminohydrolase 1	189	10PPM
Q6GQX2	Nck-associated protein 5-like	148	10PPM
Q8R007	Nectin-4	72	10PPM
P70211	Netrin receptor DCC	45	10PPM
Q9Z0E0	Neurochondrin	127	10PPM
P35802	Neuronal membrane glycoprotein M6-a	68	10PPM
Q9QXX8	Nuclear fragile X mental retardation-interacting protein 1	99	10PPM
O54998	Peptidyl-prolyl cis-trans isomerase FKBP7	100	10PPM
Q5BL07	Peroxisome biogenesis factor 1	141	10PPM
Q91YL7	PGAP2-interacting protein	53	10PPM
O70167	Phosphatidylinositol 4-phosphate 3-kinase C2 domain-containing subunit gamma	66	10PPM
O70250	Phosphoglycerate mutase 2	113	10PPM
P98199	Phospholipid-transporting ATPase ID	48	10PPM
Q8CDG1	Piwi-like protein 2	132	10PPM
P0CG49	Polyubiquitin-B	1318	10PPM
P0CG50	Polyubiquitin-C	1318	10PPM
Q9JJV2	Profilin-2	1172	10PPM
O35129	Prohibitin-2	402	10PPM
Q9QXV0	ProSAAS	113	10PPM
Q61207	Prosaposin	75	10PPM

Q8BHZ0	Protein FAM49A	149	10PPM
Q921M7	Protein FAM49B	68	10PPM
Q8BPI1	Protein kintoun	84	10PPM
Q8VHQ3	Protein phosphatase 1 regulatory inhibitor subunit 16B	105	10PPM
Q9CU24	Protein THEMIS3	47	10PPM
P35294	Ras-related protein Rab-19	293	10PPM
P24549	Retinal dehydrogenase 1	133	10PPM
Q8BRH3	Rho GTPase-activating protein 19	82	10PPM
Q8BWA8	Rho guanine nucleotide exchange factor 19	263	10PPM
Q9JLC8	Sacsin	80	10PPM
Q60988	SCL-interrupting locus protein homolog	36	10PPM
Q9CZC8	Secernin-1	251	10PPM
Q9R1T4	Septin-6	292	10PPM
P48455	Serine/threonine-protein phosphatase 2B catalytic subunit gamma isoform	82	10PPM
B2RXR6	Serine/threonine-protein phosphatase 6 regulatory ankyrin repeat subunit B	116	10PPM
Q62388	Serine-protein kinase ATM	27	10PPM
Q4VA53	Sister chromatid cohesion protein PDS5 homolog B	44	10PPM
Q3UHA3	Spatacsin	35	10PPM
P52019	Squalene monooxygenase	140	10PPM
Q8R191	Synaptogyrin-3	651	10PPM
Q9R0N5	Synaptotagmin-5	94	10PPM
Q71LX4	Talin-2	49	10PPM
A6H6E9	Tetratricopeptide repeat protein 23-like	79	10PPM
Q6PD31	Trafficking kinesin-binding protein 1	73	10PPM
Q3UR70	Transforming growth factor-beta receptor-associated protein 1	102	10PPM
P20801	Troponin C_ skeletal muscle	558	10PPM
Q7TQD2	Tubulin polymerization-promoting protein	290	10PPM
Q8CIV8	Tubulin-specific chaperone E	43	10PPM
Q00993	Tyrosine-protein kinase receptor UFO	52	10PPM
P56399	Ubiquitin carboxyl-terminal hydrolase 5	69	10PPM

P62983	Ubiquitin-40S ribosomal protein S27a	1318	10PPM
P62984	Ubiquitin-60S ribosomal protein L40	1318	10PPM
Q9DBP5	UMP-CMP kinase	92	10PPM
P63044	Vesicle-associated membrane protein 2	877	10PPM
P63024	Vesicle-associated membrane protein 3	40	10PPM
Q9WTT4	V-type proton ATPase subunit G 2	1914	10PPM
O88342	WD repeat-containing protein 1	165	10PPM
P63054	Calmodulin regulator protein PCP4	1563	50PPM
Q8VD04	GRIP1-associated protein 1	22	50PPM
Q9EQW7	Kinesin-like protein KIF13A	41	50PPM
Q4FZC9	Nesprin-3	76	50PPM
Q9Z1S3	RAS guanyl-releasing protein 1	38	50PPM

[a]Accession ID according to Uniport.org database.

Table S2. Identified proteins with different expression in cerebellum of mice exposed to 10 mg F/L vs. control group.

Accession ID[a]	Protein Name	*PLGS* Score	Fold Change
Q9CQV8	14-3-3 protein beta/alpha	1746	-0,59
P62259	14-3-3 protein epsilon	1502	-0,48
P68510	14-3-3 protein eta	1909	-0,50
P61982	14-3-3 protein gamma	2809	-0,53
O70456	14-3-3 protein sigma	1610	-0,49
P68254	14-3-3 protein theta	1750	-0,55
P63101	14-3-3 protein zeta/delta	4317	-0,52
P16330	2'_3'-cyclic-nucleotide 3'-phosphodiesterase	3402	-0,38
P52760	2-iminobutanoate/2-iminopropanoate deaminase	315	-0,40
Q60597	2-oxoglutarate dehydrogenase_ mitochondrial	67	-0,34
P61922	4-aminobutyrate aminotransferase_ mitochondrial	470	-0,52
P63038	60 kDa heat shock protein_ mitochondrial	1658	-0,40
Q8QZT1	Acetyl-CoA acetyltransferase_ mitochondrial	402	-0,51
Q99KI0	Aconitate hydratase_ mitochondrial	6506	-0,38
P68033	Actin_ alpha cardiac muscle 1	37080	-0,48
P68134	Actin_ alpha skeletal muscle	37080	-0,48
P62737	Actin_ aortic smooth muscle	34586	-0,49
P60710	Actin_ cytoplasmic 1	50515	-0,51
P63260	Actin_ cytoplasmic 2	50515	-0,51
P63268	Actin_ gamma-enteric smooth muscle	34586	-0,48
P31786	Acyl-CoA-binding protein	969	-0,50
P48962	ADP/ATP translocase 1	1773	-0,48
P51881	ADP/ATP translocase 2	1172	-0,44
Q3V132	ADP/ATP translocase 4	185	-0,49
P17182	Alpha-enolase	18993	-0,46
P46660	Alpha-internexin	1337	-0,44
P05201	Aspartate aminotransferase_ cytoplasmic	2744	-0,44
P05202	Aspartate aminotransferase_ mitochondrial	2665	-0,42
Q9CQQ7	ATP synthase F(0) complex subunit B1_ mitochondrial	577	-0,44
Q03265	ATP synthase subunit alpha_ mitochondrial	6200	-0,42
P56480	ATP synthase subunit beta_ mitochondrial	12355	-0,35

Q9DCX2	ATP synthase subunit d_ mitochondrial	1456	-0,30
Q9D3D9	ATP synthase subunit delta_ mitochondrial	1133	-0,27
P56135	ATP synthase subunit f_ mitochondrial	4503	-0,25
Q9DB20	ATP synthase subunit O_ mitochondrial	4634	-0,48
P97450	ATP synthase-coupling factor 6_ mitochondrial	987	-0,52
P47857	ATP-dependent 6-phosphofructokinase_ muscle type	242	-0,48
Q8BFZ3	Beta-actin-like protein 2	14152	-0,51
P21550	Beta-enolase	4346	-0,62
P28652	Calcium/calmodulin-dependent protein kinase type II subunit beta	873	-0,87
Q923T9	Calcium/calmodulin-dependent protein kinase type II subunit gamma	795	-0,84
Q8BH59	Calcium-binding mitochondrial carrier protein Aralar1	371	-0,50
P28651	Carbonic anhydrase-related protein	687	-0,38
Q99N28	Cell adhesion molecule 3	168	-0,50
Q9CZU6	Citrate synthase_ mitochondrial	759	-0,70
Q68FD5	Clathrin heavy chain 1	745	-0,52
P18760	Cofilin-1	3746	-0,51
P45591	Cofilin-2	890	-0,70
P12960	Contactin-1	138	-0,76
Q04447	Creatine kinase B-type	16034	-0,35
P30275	Creatine kinase U-type_ mitochondrial	2199	-0,41
Q9CZ13	Cytochrome b-c1 complex subunit 1_ mitochondrial	3968	-0,43
Q9DB77	Cytochrome b-c1 complex subunit 2_ mitochondrial	405	-0,46
Q9CR68	Cytochrome b-c1 complex subunit Rieske_ mitochondrial	751	-0,55
P00405	Cytochrome c oxidase subunit 2	2395	-0,37
P19783	Cytochrome c oxidase subunit 4 isoform 1_ mitochondrial	979	-0,39
P12787	Cytochrome c oxidase subunit 5A_ mitochondrial	2891	-0,38
P43024	Cytochrome c oxidase subunit 6A1_ mitochondrial	1081	-0,35
P56391	Cytochrome c oxidase subunit 6B1	615	-0,64
P62897	Cytochrome c_ somatic	336	-0,68
Q9D0M3	Cytochrome c1_ heme protein_ mitochondrial	1841	-0,44
Q9JHU4	Cytoplasmic dynein 1 heavy chain 1	61	-0,27
Q91V12	Cytosolic acyl coenzyme A thioester hydrolase	243	-0,62
P31001	Desmin	357	-0,44
O08749	Dihydrolipoyl dehydrogenase_ mitochondrial	352	-0,63
Q8BMF4	Dihydrolipoyllysine-residue acetyltransferase component of pyruvate dehydrogenase complex_ mitochondrial	286	-0,44
P97427	Dihydropyrimidinase-related protein 1	2802	-0,53

O08553	Dihydropyrimidinase-related protein 2	7819	-0,45
Q62188	Dihydropyrimidinase-related protein 3	1426	-0,52
O35098	Dihydropyrimidinase-related protein 4	179	-0,49
P39053	Dynamin-1	2982	-0,59
P39054	Dynamin-2	167	-0,79
Q8BZ98	Dynamin-3	153	-0,76
O88343	Electrogenic sodium bicarbonate cotransporter 1	87	-0,51
P10126	Elongation factor 1-alpha 1	1234	-0,39
P62631	Elongation factor 1-alpha 2	1889	-0,43
Q62420	Endophilin-A1	1559	-0,74
P20029	Endoplasmic reticulum chaperone BiP	1442	-0,66
P54754	Ephrin type-B receptor 3	87	-0,34
P56564	Excitatory amino acid transporter 1	1245	-0,37
P43006	Excitatory amino acid transporter 2	258	1,58
P05064	Fructose-bisphosphate aldolase A	6921	-0,39
P05063	Fructose-bisphosphate aldolase C	5303	-0,35
P97807	Fumarate hydratase_ mitochondrial	398	-0,55
P17183	Gamma-enolase	12105	-0,50
P06745	Glucose-6-phosphate isomerase	2193	-0,48
P26443	Glutamate dehydrogenase 1_ mitochondrial	1225	-0,54
P15105	Glutamine synthetase	1524	-0,56
P10649	Glutathione S-transferase Mu 1	1413	-0,46
P15626	Glutathione S-transferase Mu 2	430	-0,44
P48774	Glutathione S-transferase Mu 5	115	-0,52
Q80W21	Glutathione S-transferase Mu 7	430	-0,43
P46425	Glutathione S-transferase P 2	157	-0,66
P16858	Glyceraldehyde-3-phosphate dehydrogenase	19433	-0,36
Q64467	Glyceraldehyde-3-phosphate dehydrogenase_ testis-specific	1100	-0,62
Q64521	Glycerol-3-phosphate dehydrogenase_ mitochondrial	100	-0,46
Q8CI94	Glycogen phosphorylase_ brain form	194	-0,32
Q9ET01	Glycogen phosphorylase_ liver form	82	-0,36
Q9WUB3	Glycogen phosphorylase_ muscle form	110	-0,29
B2RSH2	Guanine nucleotide-binding protein G(i) subunit alpha-1	668	-0,61
P08752	Guanine nucleotide-binding protein G(i) subunit alpha-2	668	-0,61
P62874	Guanine nucleotide-binding protein G(I)/G(S)/G(T) subunit beta-1 3	944	-0,84
P62880	Guanine nucleotide-binding protein G(I)/G(S)/G(T) subunit beta-2	697	-0,83

Q61011	Guanine nucleotide-binding protein G(I)/G(S)/G(T) subunit beta-3	187	-0,54
Q9DC51	Guanine nucleotide-binding protein G(k) subunit alpha	668	-0,68
P18872	Guanine nucleotide-binding protein G(o) subunit alpha	2486	-0,56
Q8CGK7	Guanine nucleotide-binding protein G(olf) subunit alpha	676	-0,70
P63094	Guanine nucleotide-binding protein G(s) subunit alpha isoforms short	668	-0,73
P20612	Guanine nucleotide-binding protein G(t) subunit alpha-1	668	-0,73
Q3V3I2	Guanine nucleotide-binding protein G(t) subunit alpha-3	668	-0,73
P27600	Guanine nucleotide-binding protein subunit alpha-12	724	-0,73
P27601	Guanine nucleotide-binding protein subunit alpha-13	724	-0,74
P29387	Guanine nucleotide-binding protein subunit beta-4	264	-0,69
Q61696	Heat shock 70 kDa protein 1A	2624	-0,53
P17879	Heat shock 70 kDa protein 1B	2624	-0,51
P16627	Heat shock 70 kDa protein 1-like	2626	-0,54
P48722	Heat shock 70 kDa protein 4L	541	-0,25
P63017	Heat shock cognate 71 kDa protein	9714	-0,38
Q9CQN1	Heat shock protein 75 kDa_ mitochondrial	2810	-0,40
P07901	Heat shock protein HSP 90-alpha	4828	-0,45
P11499	Heat shock protein HSP 90-beta	5178	-0,51
P17156	Heat shock-related 70 kDa protein 2	3330	-0,40
P01942	Hemoglobin subunit alpha	12471	-0,42
P02088	Hemoglobin subunit beta-1	20421	-0,42
P02089	Hemoglobin subunit beta-2	7406	-0,42
P06467	Hemoglobin subunit zeta	95	-0,28
O88569	Heterogeneous nuclear ribonucleoproteins A2/B1	341	-0,42
C0HKE1	Histone H2A type 1-B	20058	-0,64
C0HKE2	Histone H2A type 1-C	20058	-0,65
C0HKE3	Histone H2A type 1-D	20058	-0,64
C0HKE4	Histone H2A type 1-E	20058	-0,64
Q8CGP5	Histone H2A type 1-F	20058	-0,65
C0HKE5	Histone H2A type 1-G	20058	-0,64
Q8CGP6	Histone H2A type 1-H	20058	-0,64
C0HKE6	Histone H2A type 1-I	20058	-0,65
Q8CGP7	Histone H2A type 1-K	20058	-0,63
C0HKE7	Histone H2A type 1-N	20058	-0,65
C0HKE8	Histone H2A type 1-O	20058	-0,64
C0HKE9	Histone H2A type 1-P	20058	-0,64

Q6GSS7	Histone H2A type 2-A	20058	-0,65
Q64523	Histone H2A type 2-C	20058	-0,64
Q8BFU2	Histone H2A type 3	20058	-0,64
Q8R1M2	Histone H2A.J	20058	-0,65
Q3THW5	Histone H2A.V	3856	-0,50
P0C0S6	Histone H2A.Z	3856	-0,51
P27661	Histone H2AX	3856	-0,57
P70696	Histone H2B type 1-A	1309	-0,16
Q64475	Histone H2B type 1-B	16744	-0,50
Q6ZWY9	Histone H2B type 1-C/E/G	16744	-0,50
P10853	Histone H2B type 1-F/J/L	16744	-0,50
Q64478	Histone H2B type 1-H	16744	-0,52
Q8CGP1	Histone H2B type 1-K	16744	-0,50
P10854	Histone H2B type 1-M	16744	-0,50
Q8CGP2	Histone H2B type 1-P	16744	-0,50
Q64525	Histone H2B type 2-B	16744	-0,50
Q64524	Histone H2B type 2-E	11290	-0,53
Q9D2U9	Histone H2B type 3-A	11290	-0,53
Q8CGP0	Histone H2B type 3-B	11290	-0,53
P62806	Histone H4	6545	-0,86
P11881	Inositol 1_4_5-trisphosphate receptor type 1	398	-0,39
Q9D6R2	Isocitrate dehydrogenase [NAD] subunit alpha_ mitochondrial	143	-0,60
Q61595	Kinectin	273	-0,15
P06151	L-lactate dehydrogenase A chain	1657	-0,68
P16125	L-lactate dehydrogenase B chain	3759	-0,45
P00342	L-lactate dehydrogenase C chain	1423	-0,57
P14152	Malate dehydrogenase_ cytoplasmic	3939	-0,47
P08249	Malate dehydrogenase_ mitochondrial	17070	-0,48
P20357	Microtubule-associated protein 2	88	4,39
P04370	Myelin basic protein	8671	-0,47
P60202	Myelin proteolipid protein	7221	-0,45
Q8VDQ8	NAD-dependent protein deacetylase sirtuin-2	96	-0,47
Q91YT0	NADH dehydrogenase [ubiquinone] flavoprotein 1_ mitochondrial	578	-0,34
Q9D6J6	NADH dehydrogenase [ubiquinone] flavoprotein 2_ mitochondrial	766	-0,54
Q9DCT2	NADH dehydrogenase [ubiquinone] iron-sulfur protein 3_ mitochondrial	247	-0,47
Q91VD9	NADH-ubiquinone oxidoreductase 75 kDa subunit_ mitochondrial	264	-0,53

P13595	Neural cell adhesion molecule 1	138	-0,54
P19246	Neurofilament heavy polypeptide	359	-0,42
P08551	Neurofilament light polypeptide	2082	-0,34
P08553	Neurofilament medium polypeptide	1126	-0,36
P06837	Neuromodulin	194	1,80
P15532	Nucleoside diphosphate kinase A	1453	-0,70
P17742	Peptidyl-prolyl cis-trans isomerase A	9325	-0,59
P15331	Peripherin	44	-0,34
P35700	Peroxiredoxin-1	773	-0,58
Q61171	Peroxiredoxin-2	1174	-0,51
P99029	Peroxiredoxin-5_ mitochondrial	5504	-0,38
O08709	Peroxiredoxin-6	638	-0,45
Q8VEM8	Phosphate carrier protein_ mitochondrial	368	-0,36
P70296	Phosphatidylethanolamine-binding protein 1	1662	-0,64
P09411	Phosphoglycerate kinase 1	4682	-0,45
P09041	Phosphoglycerate kinase 2	2871	-0,52
Q9DBJ1	Phosphoglycerate mutase 1	2910	-0,45
Q64436	Potassium-transporting ATPase alpha chain 1	200	-0,42
Q9Z1W8	Potassium-transporting ATPase alpha chain 2	619	-0,33
P62962	Profilin-1	325	-0,70
Q99LX0	Protein/nucleic acid deglycase DJ-1	1124	-0,66
P12660	Purkinje cell protein 2	1032	-0,42
Q9D051	Pyruvate dehydrogenase E1 component subunit beta_ mitochondrial	461	-0,65
P53657	Pyruvate kinase PKLR	1173	-0,50
P52480	Pyruvate kinase PKM	14446	-0,46
P50396	Rab GDP dissociation inhibitor alpha	2114	-0,53
Q61598	Rab GDP dissociation inhibitor beta	1800	-0,51
Q9DD03	Ras-related protein Rab-13	484	1,51
P63011	Ras-related protein Rab-3A	2203	-0,76
Q8C650	Septin-10	310	-0,59
Q8C1B7	Septin-11	397	-0,61
Q9DA97	Septin-14	310	-0,60
O55131	Septin-7	627	-0,61
P63328	Serine/threonine-protein phosphatase 2B catalytic subunit alpha isoform	221	1,62
P48453	Serine/threonine-protein phosphatase 2B catalytic subunit beta isoform	141	1,58
P07724	Serum albumin	519	-0,43

Q8VDN2	Sodium/potassium-transporting ATPase subunit alpha-1	2369	-0,38
Q6PIE5	Sodium/potassium-transporting ATPase subunit alpha-2	2503	-0,35
Q6PIC6	Sodium/potassium-transporting ATPase subunit alpha-3	3167	-0,38
Q9WV27	Sodium/potassium-transporting ATPase subunit alpha-4	747	-0,32
P14094	Sodium/potassium-transporting ATPase subunit beta-1	5250	-0,38
P14231	Sodium/potassium-transporting ATPase subunit beta-2	253	-0,35
P16546	Spectrin alpha chain_ non-erythrocytic 1	262	-0,57
Q62261	Spectrin beta chain_ non-erythrocytic 1	115	-0,81
P38647	Stress-70 protein_ mitochondrial	102	-0,35
Q9Z2I9	Succinate--CoA ligase [ADP-forming] subunit beta_ mitochondrial	296	-0,49
Q8BWF0	Succinate-semialdehyde dehydrogenase_ mitochondrial	299	-0,39
P09671	Superoxide dismutase [Mn]_ mitochondrial	161	-0,36
O88935	Synapsin-1	465	-0,63
Q64332	Synapsin-2	410	-0,70
Q62277	Synaptophysin	281	-0,65
P60879	Synaptosomal-associated protein 25	648	-0,42
P46096	Synaptotagmin-1	213	1,92
P61264	Syntaxin-1B	455	-0,53
O08599	Syntaxin-binding protein 1	8818	-0,39
P01831	Thy-1 membrane glycoprotein	555	1,51
P42669	Transcriptional activator protein Pur-alpha	178	-0,50
Q01853	Transitional endoplasmic reticulum ATPase	278	-0,53
P40142	Transketolase	204	-0,58
P17751	Triosephosphate isomerase	4965	-0,45
Q3UX10	Tubulin alpha chain-like 3	577	-0,59
P68369	Tubulin alpha-1A chain	25243	-0,50
P05213	Tubulin alpha-1B chain	26960	-0,49
P68373	Tubulin alpha-1C chain	24678	-0,49
P05214	Tubulin alpha-3 chain	20335	-0,41
P68368	Tubulin alpha-4A chain	24861	-0,44
Q9JJZ2	Tubulin alpha-8 chain	17875	-0,41
A2AQ07	Tubulin beta-1 chain	1569	-0,41
Q7TMM9	Tubulin beta-2A chain	54982	-0,45
Q9CWF2	Tubulin beta-2B chain	54924	-0,45
Q9ERD7	Tubulin beta-3 chain	26347	-0,41
Q9D6F9	Tubulin beta-4A chain	45683	-0,41

P68372	Tubulin beta-4B chain	52744	-0,42
P99024	Tubulin beta-5 chain	54945	-0,41
Q922F4	Tubulin beta-6 chain	14581	-0,43
Q9R0P9	Ubiquitin carboxyl-terminal hydrolase isozyme L1	735	-0,53
Q02053	Ubiquitin-like modifier-activating enzyme 1	187	-0,58
P35917	Vascular endothelial growth factor receptor 3	84	-0,33
E9PYH0	Versican core protein	223	-0,52
Q62059	Versican core protein	223	-0,53
P20152	Vimentin	664	-0,47
Q60932	Voltage-dependent anion-selective channel protein 1	1177	-0,46
Q60930	Voltage-dependent anion-selective channel protein 2	1067	-0,48
Q60931	Voltage-dependent anion-selective channel protein 3	157	-0,49
Q8CC88	von Willebrand factor A domain-containing protein 8	68	2,36
P50516	V-type proton ATPase catalytic subunit A	1615	-0,58
P50518	V-type proton ATPase subunit E 1	392	1,54

The identified proteins are organized according to the alphabetical order. Relative differential expression is indicated by positive value, when the protein is upregulated, and by negative values (−), when the protein is downregulated in the comparison between groups. aIdentification is based on protein ID from UniProt protein database (http://www.uniprot.org/).

Table S3. Identified proteins with different expression in cerebellum of mice exposed to 50 mg F/L vs. control group.

Accession ID[a]	Protein Name	*PLGS* Score	Fold Change
Q64433	10 kDa heat shock protein_ mitochondrial	2731	-0,38
Q9CQV8	14-3-3 protein beta/alpha	1746	-0,39
P62259	14-3-3 protein epsilon	1502	-0,38
P68510	14-3-3 protein eta	1909	-0,38
P61982	14-3-3 protein gamma	2809	-0,39
O70456	14-3-3 protein sigma	1610	-0,39
P68254	14-3-3 protein theta	1750	-0,39
P63101	14-3-3 protein zeta/delta	4317	-0,40
P16330	2'_3'-cyclic-nucleotide 3'-phosphodiesterase	3402	-0,32
P63038	60 kDa heat shock protein_ mitochondrial	1658	-0,34
Q99KI0	Aconitate hydratase_ mitochondrial	6506	-0,32
P68033	Actin_ alpha cardiac muscle 1	37080	-0,43
P68134	Actin_ alpha skeletal muscle	37080	-0,42
P62737	Actin_ aortic smooth muscle	34586	-0,42
P60710	Actin_ cytoplasmic 1	50515	-0,43
P63260	Actin_ cytoplasmic 2	50515	-0,42
P63268	Actin_ gamma-enteric smooth muscle	34586	-0,41
P31786	Acyl-CoA-binding protein	969	-0,40
P48962	ADP/ATP translocase 1	1773	-0,38
P51881	ADP/ATP translocase 2	1172	-0,32
Q3V132	ADP/ATP translocase 4	185	-0,33
P17182	Alpha-enolase	18993	-0,34
P46660	Alpha-internexin	1337	-0,44
O55042	Alpha-synuclein	338	-0,45
P05201	Aspartate aminotransferase_ cytoplasmic	2744	-0,31
P05202	Aspartate aminotransferase_ mitochondrial	2665	-0,33
Q9CQQ7	ATP synthase F(0) complex subunit B1_ mitochondrial	577	-0,33
Q03265	ATP synthase subunit alpha_ mitochondrial	6200	-0,39
P56480	ATP synthase subunit beta_ mitochondrial	12355	-0,33
Q9DCX2	ATP synthase subunit d_ mitochondrial	1456	-0,21
P56135	ATP synthase subunit f_ mitochondrial	4503	-0,28

Q9DB20	ATP synthase subunit O_ mitochondrial	4634	-0,41
P12382	ATP-dependent 6-phosphofructokinase_ liver type	39	-0,49
P47857	ATP-dependent 6-phosphofructokinase_ muscle type	242	-0,30
Q9WUA3	ATP-dependent 6-phosphofructokinase_ platelet type	74	-0,38
Q8BFZ3	Beta-actin-like protein 2	14152	-0,44
Q91ZZ3	Beta-synuclein	1047	-0,48
P12658	Calbindin	128	-0,62
P0DP26	Calmodulin-1	375	-0,71
P0DP27	Calmodulin-2	375	-0,76
P0DP28	Calmodulin-3	375	-0,73
P28651	Carbonic anhydrase-related protein	687	-0,36
Q68FD5	Clathrin heavy chain 1	745	-0,33
P18760	Cofilin-1	3746	-0,42
P45591	Cofilin-2	890	-0,50
Q04447	Creatine kinase B-type	16034	-0,40
P30275	Creatine kinase U-type_ mitochondrial	2199	-0,28
Q9CZ13	Cytochrome b-c1 complex subunit 1_ mitochondrial	3968	-0,34
Q9DB77	Cytochrome b-c1 complex subunit 2_ mitochondrial	405	-0,34
Q9CR68	Cytochrome b-c1 complex subunit Rieske_ mitochondrial	751	-0,39
P00405	Cytochrome c oxidase subunit 2	2395	-0,30
P19783	Cytochrome c oxidase subunit 4 isoform 1_ mitochondrial	979	-0,30
P12787	Cytochrome c oxidase subunit 5A_ mitochondrial	2891	-0,25
Q62425	Cytochrome c oxidase subunit NDUFA4	1401	-0,41
P31001	Desmin	357	-0,48
P97427	Dihydropyrimidinase-related protein 1	2802	-0,35
O08553	Dihydropyrimidinase-related protein 2	7819	-0,35
Q62188	Dihydropyrimidinase-related protein 3	1426	-0,35
O35098	Dihydropyrimidinase-related protein 4	179	-0,23
P39053	Dynamin-1	2982	-0,35
P39054	Dynamin-2	167	-0,42
Q8BZ98	Dynamin-3	153	-0,53
P10126	Elongation factor 1-alpha 1	1234	-0,31
P62631	Elongation factor 1-alpha 2	1889	-0,30
P20029	Endoplasmic reticulum chaperone BiP	1442	-0,46
P56564	Excitatory amino acid transporter 1	1245	-0,34
P05064	Fructose-bisphosphate aldolase A	6921	-0,32

P05063	Fructose-bisphosphate aldolase C	5303	-0,34
P17183	Gamma-enolase	12105	-0,36
P26443	Glutamate dehydrogenase 1_ mitochondrial	1225	-0,36
P15105	Glutamine synthetase	1524	-0,43
P10649	Glutathione S-transferase Mu 1	1413	-0,37
P15626	Glutathione S-transferase Mu 2	430	-0,29
Q80W21	Glutathione S-transferase Mu 7	430	-0,29
P16858	Glyceraldehyde-3-phosphate dehydrogenase	19433	-0,41
Q64467	Glyceraldehyde-3-phosphate dehydrogenase_ testis-specific	1100	-0,52
B2RSH2	Guanine nucleotide-binding protein G(i) subunit alpha-1	668	-0,46
P08752	Guanine nucleotide-binding protein G(i) subunit alpha-2	668	-0,47
P62874	Guanine nucleotide-binding protein G(I)/G(S)/G(T) subunit beta-1	944	-0,49
P62880	Guanine nucleotide-binding protein G(I)/G(S)/G(T) subunit beta-2	697	-0,49
Q61011	Guanine nucleotide-binding protein G(I)/G(S)/G(T) subunit beta-3	187	-0,63
Q9DC51	Guanine nucleotide-binding protein G(k) subunit alpha	668	-0,45
P18872	Guanine nucleotide-binding protein G(o) subunit alpha	2486	-0,42
Q8CGK7	Guanine nucleotide-binding protein G(olf) subunit alpha	676	-0,48
P63094	Guanine nucleotide-binding protein G(s) subunit alpha isoforms short	668	-0,48
Q6R0H7	Guanine nucleotide-binding protein G(s) subunit alpha isoforms XLas	668	-0,47
P20612	Guanine nucleotide-binding protein G(t) subunit alpha-1	668	-0,48
P50149	Guanine nucleotide-binding protein G(t) subunit alpha-2	668	-0,48
Q3V3I2	Guanine nucleotide-binding protein G(t) subunit alpha-3	668	-0,48
P27600	Guanine nucleotide-binding protein subunit alpha-12	724	-0,47
P27601	Guanine nucleotide-binding protein subunit alpha-13	724	-0,48
P29387	Guanine nucleotide-binding protein subunit beta-4	264	-0,69
Q61696	Heat shock 70 kDa protein 1A	2624	-0,39
P17879	Heat shock 70 kDa protein 1B	2624	-0,39
P16627	Heat shock 70 kDa protein 1-like	2626	-0,40
P63017	Heat shock cognate 71 kDa protein	9714	-0,33
Q9CQN1	Heat shock protein 75 kDa_ mitochondrial	2810	-0,28
P07901	Heat shock protein HSP 90-alpha	4828	-0,28
P11499	Heat shock protein HSP 90-beta	5178	-0,31
P17156	Heat shock-related 70 kDa protein 2	3330	-0,35
P01942	Hemoglobin subunit alpha	12471	-0,40
P02088	Hemoglobin subunit beta-1	20421	-0,35
P02089	Hemoglobin subunit beta-2	7406	-0,33

P06467	Hemoglobin subunit zeta	95	-0,36
P70349	Histidine triad nucleotide-binding protein 1	1124	-0,29
C0HKE1	Histone H2A type 1-B	20058	-0,39
C0HKE2	Histone H2A type 1-C	20058	-0,39
C0HKE3	Histone H2A type 1-D	20058	-0,39
C0HKE4	Histone H2A type 1-E	20058	-0,39
Q8CGP5	Histone H2A type 1-F	20058	-0,39
C0HKE5	Histone H2A type 1-G	20058	-0,39
Q8CGP6	Histone H2A type 1-H	20058	-0,39
C0HKE6	Histone H2A type 1-I	20058	-0,39
Q8CGP7	Histone H2A type 1-K	20058	-0,39
C0HKE7	Histone H2A type 1-N	20058	-0,39
C0HKE8	Histone H2A type 1-O	20058	-0,39
C0HKE9	Histone H2A type 1-P	20058	-0,39
Q6GSS7	Histone H2A type 2-A	20058	-0,39
Q64523	Histone H2A type 2-C	20058	-0,39
Q8BFU2	Histone H2A type 3	20058	-0,39
Q8R1M2	Histone H2A.J	20058	-0,39
Q3THW5	Histone H2A.V	3856	-0,45
P0C0S6	Histone H2A.Z	3856	-0,44
P27661	Histone H2AX	3856	-0,44
P70696	Histone H2B type 1-A	1309	-0,46
Q64475	Histone H2B type 1-B	16744	-0,38
Q6ZWY9	Histone H2B type 1-C/E/G	16744	-0,38
P10853	Histone H2B type 1-F/J/L	16744	-0,38
Q64478	Histone H2B type 1-H	16744	-0,38
Q8CGP1	Histone H2B type 1-K	16744	-0,38
P10854	Histone H2B type 1-M	16744	-0,38
Q8CGP2	Histone H2B type 1-P	16744	-0,38
Q64525	Histone H2B type 2-B	16744	-0,38
Q64524	Histone H2B type 2-E	11290	-0,39
Q9D2U9	Histone H2B type 3-A	11290	-0,39
Q8CGP0	Histone H2B type 3-B	11290	-0,39
P68433	Histone H3.1	1073	-0,39
P84228	Histone H3.2	1073	-0,39
P84244	Histone H3.3	1073	-0,41

P02301	Histone H3.3C	1073	-0,41
P62806	Histone H4	6545	-0,59
Q9D6R2	Isocitrate dehydrogenase [NAD] subunit alpha_ mitochondrial	143	-0,43
P70404	Isocitrate dehydrogenase [NAD] subunit gamma 1_ mitochondrial	154	-0,37
P16125	L-lactate dehydrogenase B chain	3759	-0,36
P14152	Malate dehydrogenase_ cytoplasmic	3939	-0,32
P08249	Malate dehydrogenase_ mitochondrial	17070	-0,43
P04370	Myelin basic protein	8671	-0,46
P60202	Myelin proteolipid protein	7221	-0,50
Q61879	Myosin-10	125	-0,35
P19246	Neurofilament heavy polypeptide	359	-0,41
P08551	Neurofilament light polypeptide	2082	-0,38
P08553	Neurofilament medium polypeptide	1126	-0,37
P17742	Peptidyl-prolyl cis-trans isomerase A	9325	-0,40
P35700	Peroxiredoxin-1	773	-0,26
P99029	Peroxiredoxin-5_ mitochondrial	5504	-0,33
O08709	Peroxiredoxin-6	638	-0,25
Q8VEM8	Phosphate carrier protein_ mitochondrial	368	-0,27
P70296	Phosphatidylethanolamine-binding protein 1	1662	-0,40
P09411	Phosphoglycerate kinase 1	4682	-0,25
P09041	Phosphoglycerate kinase 2	2871	-0,35
Q9DBJ1	Phosphoglycerate mutase 1	2910	-0,25
Q64436	Potassium-transporting ATPase alpha chain 1	200	-0,35
Q9Z1W8	Potassium-transporting ATPase alpha chain 2	619	-0,41
Q924A2	Protein capicua homolog	53	-0,55
Q99LX0	Protein/nucleic acid deglycase DJ-1	1124	-0,42
P12660	Purkinje cell protein 2	1032	-0,42
Q9D051	Pyruvate dehydrogenase E1 component subunit beta_ mitochondrial	461	-0,38
P53657	Pyruvate kinase PKLR	1173	-0,38
P52480	Pyruvate kinase PKM	14446	-0,39
P50396	Rab GDP dissociation inhibitor alpha	2114	-0,34
Q61598	Rab GDP dissociation inhibitor beta	1800	-0,32
P61027	Ras-related protein Rab-10	1483	-0,36
P35283	Ras-related protein Rab-12	1178	-0,36
Q91V41	Ras-related protein Rab-14	1378	-0,34
Q8K386	Ras-related protein Rab-15	1436	-0,34

P62821	Ras-related protein Rab-1A	1693	-0,35
Q9D1G1	Ras-related protein Rab-1B	1636	-0,35
Q504M8	Ras-related protein Rab-26	1178	-0,34
Q923S9	Ras-related protein Rab-30	1178	-0,34
O35963	Ras-related protein Rab-33B	1017	-0,35
Q6PHN9	Ras-related protein Rab-35	1473	-0,35
Q9JKM7	Ras-related protein Rab-37	1178	-0,35
Q8BHD0	Ras-related protein Rab-39A	1070	-0,36
Q8BHC1	Ras-related protein Rab-39B	1232	-0,35
P63011	Ras-related protein Rab-3A	2203	-0,35
Q9CZT8	Ras-related protein Rab-3B	1632	-0,39
P62823	Ras-related protein Rab-3C	1632	-0,38
P35276	Ras-related protein Rab-3D	1608	-0,40
Q8CG50	Ras-related protein Rab-43	1178	-0,38
P56371	Ras-related protein Rab-4A	1178	-0,38
Q91ZR1	Ras-related protein Rab-4B	1178	-0,35
P35279	Ras-related protein Rab-6A	1070	-0,34
P61294	Ras-related protein Rab-6B	1070	-0,34
P55258	Ras-related protein Rab-8A	1624	-0,33
P61028	Ras-related protein Rab-8B	1624	-0,35
P07724	Serum albumin	519	-0,42
Q8VDN2	Sodium/potassium-transporting ATPase subunit alpha-1	2369	-0,34
Q6PIE5	Sodium/potassium-transporting ATPase subunit alpha-2	2503	-0,34
Q6PIC6	Sodium/potassium-transporting ATPase subunit alpha-3	3167	-0,36
Q9WV27	Sodium/potassium-transporting ATPase subunit alpha-4	747	-0,37
P14094	Sodium/potassium-transporting ATPase subunit beta-1	5250	-0,30
P14231	Sodium/potassium-transporting ATPase subunit beta-2	253	-0,21
P16546	Spectrin alpha chain_ non-erythrocytic 1	262	-0,39
Q62261	Spectrin beta chain_ non-erythrocytic 1	115	-0,41
P08228	Superoxide dismutase [Cu-Zn]	1087	-0,24
O88935	Synapsin-1	465	-0,33
Q64332	Synapsin-2	410	-0,37
Q62277	Synaptophysin	281	-0,29
P60879	Synaptosomal-associated protein 25	648	-0,29
P61264	Syntaxin-1B	455	-0,39
O08599	Syntaxin-binding protein 1	8818	-0,34

P01831	Thy-1 membrane glycoprotein	555	-0,46
P17751	Triosephosphate isomerase	4965	-0,32
Q3UX10	Tubulin alpha chain-like 3	577	-0,61
P68369	Tubulin alpha-1A chain	25243	-0,45
P05213	Tubulin alpha-1B chain	26960	-0,45
P68373	Tubulin alpha-1C chain	24678	-0,45
P05214	Tubulin alpha-3 chain	20335	-0,43
P68368	Tubulin alpha-4A chain	24861	-0,43
Q9JJZ2	Tubulin alpha-8 chain	17875	-0,39
A2AQ07	Tubulin beta-1 chain	1569	-0,43
Q7TMM9	Tubulin beta-2A chain	54982	-0,41
Q9CWF2	Tubulin beta-2B chain	54924	-0,41
Q9ERD7	Tubulin beta-3 chain	26347	-0,42
Q9D6F9	Tubulin beta-4A chain	45683	-0,37
P68372	Tubulin beta-4B chain	52744	-0,39
P99024	Tubulin beta-5 chain	54945	-0,39
Q922F4	Tubulin beta-6 chain	14581	-0,41
P46460	Vesicle-fusing ATPase	302	-0,36
P20152	Vimentin	664	-0,44
Q60932	Voltage-dependent anion-selective channel protein 1	1177	-0,38
Q60930	Voltage-dependent anion-selective channel protein 2	1067	-0,36
Q60931	Voltage-dependent anion-selective channel protein 3	157	-0,46
P50516	V-type proton ATPase catalytic subunit A	1615	-0,28
P62814	V-type proton ATPase subunit B_ brain isoform	428	-0,38

The identified proteins are organized according to the alphabetical order. Relative differential expression is indicated by positive value, when the protein is upregulated, and by negative values (−), when the protein is downregulated in the comparison between groups. aIdentification is based on protein ID from UniProt protein database (http://www.uniprot.org/).
